# A data-driven geographic information system and machine learning based multi-criteria framework for strategic wind power plant siting

**DOI:** 10.1038/s41598-026-51265-9

**Published:** 2026-05-10

**Authors:** Tewodros Gera Workineh, Getachew Biru Worku, Sharad Kumar Singh, Lokesh Kumar Dewangan, Habtamu Asresahagn Mebrat, Tassew Tadiwos Zewdie

**Affiliations:** 1 Bahir Dar University, Bahir Dar, Ethiopia; 2 University of Rwanda, Kigali, Rwanda; 3 Indian Institute of Technology Indore, Indore, India

**Keywords:** Data envelopment analysis, Fuzzy analytic hierarchy process, Geographic information system, Multi-criteria decision making, Wind farm siting, Engineering, Environmental sciences, Environmental social sciences, Mathematics and computing

## Abstract

Wind energy site selection requires robust frameworks that simultaneously address expert uncertainty, objective efficiency screening, and predictive capability beyond sampled locations. This study presents an integrated framework for strategic wind farm site selection in Ethiopia’s Amhara Region by combining Fuzzy Analytic Hierarchy Process (FAHP), efficiency analysis, and predictive modelling to overcome the limitations of static GIS approaches. The FAHP stage incorporates expert judgment through fuzzy triangular numbers to weight six variables such as wind speed, slope, elevation, distance to transmission lines, distance to roads, and land-use/land-cover to achieve a consistency ratio of 0.0175 with wind speed emerging as the dominant factor of 0.4211 weights in order to generate a spatial suitability surface and extract candidate high-potential areas. High-potential zones identified by FAHP are then evaluated as decision-making units using input-oriented Data Envelopment Analysis (DEA) models. DEA efficiency screening identifies North Shewa as frontier-efficient zone (CCR = BCC = SBM = 1.0), which contributes 32.25% of regional suitable land. Finally, machine learning (ML) models (Random Forest (RF), Support Vector Machine (SVM) and Extreme Gradient Boost (XGBoost)) are trained on 1.5 million sampled pixels from the 31-million-cell feature space to learn generalizable suitability functions. Random Forest achieved optimal performance with RMSE of 0.2981 and R^2^ of 0.8145 in regression and F1-score of 0.9207 and accuracy of 0.9237 in classification to delineates 1,698 km^2^ of high-priority corridors within North Shewa for immediate wind farm deployment. Independent validation through five different sources that includes 250 MW of Debre Birhan under Ethiopian Ministry of Finance private public project pipeline to confirm North Shewa as the highest-potential development corridor. This framework advances wind energy planning by integrating subjective weighting, objective efficiency analysis, and predictive modelling into a unified strategic decision-support system applicable for wind energy planning in data-scarce region.

## Introduction

Renewable energy has become an essential pillar of modern society. It plays a vital role in socio-economic development in response to global warming, air pollution, and other environmental challenges caused by fossil fuels^1^. In this regard, wind energy stands out at high position for the last five decades among renewable energy resources due to its maturity, economic viability, operational flexibility, and strong industrial growth^1^. Wind energy represents a robust and dependable resource that can complement other renewable sources to support a sustainable global energy future^2^.

Reports from the IEA and WWEA indicate that global installed wind power capacity had reached 1.1 TW by 2024 and contributes about 10% of total electricity generation worldwide^2,3^. China leads the global wind energy market, followed by the United States and India^3^. Wind power is projected to become the second-largest contributor to global renewable electricity generation surpassing hydropower by 2030 while solar PV will claim the leading position^2^. Despite Africa’s estimated technical wind potential of approximately 59 TW, the continent currently has only 9 GW of installed wind capacity that represent less than 1% of the global total^1^ and less than 0.015% of the estimated potential. Morocco, Egypt, South Africa, Ethiopia, and Kenya are the leading African countries in terms of wind power deployment^3,4^.

Although growth has been rapid worldwide, the successful deployment of wind energy is not solely determined by resource availability alone. Its economic viability depends on selecting suitable locations that balance technical, environmental, social, and financial considerations^5^. While wind turbines can be constructed within a relatively short timeframe, their high initial investment costs make optimal site selection essential for ensuring sustainable and cost-effective wind energy development by accounting for economic, technical, environmental, and social criteria^5^. Therefore Multi-criteria decision-making (MCDM) methods have been applied by different researchers for wind farm site suitability analysis, where MCDM encompasses multi-attribute approaches that evaluate discrete alternatives across predefined criteria, including Analytical Hierarchical process (AHP), Technique for Order Preference by Similarity to Ideal Solution (TOPSIS), Elimination and Choice Expressing Reality (ELECTRE), Preference Ranking Organization METHod for Enrichment Evaluations (PROMETHEE), and VlseKriterijumska Optimizacija I Kompromisno Resenje (VIKOR)^6,8^. In contrast, Multi-Objective Decision Making (MODM) techniques address continuous decision spaces through optimization-based approaches using optimization-based goal programming and evolutionary algorithms^6,7^. AHP remains the most frequently adopted technique in wind farm planning among MCDM techniques due to its structured pairwise comparison and consistency ratio verification, though it exhibits limitations related to judgment subjectivity, vagueness, and inconsistency^6–10^. This motivates the evolution toward Fuzzy analytical hierarchical process (FAHP) that incorporates fuzzy logic to handle imprecise and uncertain expert evaluations^11–18^. To address remaining shortcomings, hybrid Data Envelopment Analysis (DEA) and MCDM frameworks integrate efficiency analysis with subjective weighting to enhance decision robustness under uncertainty. DEA evaluates alternatives or sites as “decision-making units” (DMUs) by assessing multiple inputs variables such as cost, land use, etc. and outputs variables like energy yield, capacity factor, suitable area etc. simultaneously without requiring you to pre-assign weights. This weight-free approach calculates optimal weights endogenously for each DMU by maximizing its relative efficiency score against the performance of all other units and thereby reducing the subjectivity inherent in weight-assignment methods like AHP or FAHP^19,20^. DEA needs relatively complete, quantitative data for all inputs/outputs and a minimum number of alternatives. Missing or noisy data for key indicators can severely affect results of DEA, while AHP can more easily mix qualitative judgments and limited quantitative data^20^. Even though combining DEA and FAHP helps capture both subjective and objective judgments, this hybrid approach still lacks predictive power.

Despite extensive applications of FAHP and DEA in renewable energy site selection and performance evaluation, the existing hybrid models remain static and limited to ranking outcomes without the capability of predictive decision support. These traditional frameworks cannot generalize to new unseen sites beyond the initial dataset since it is static in nature. The framework also struggles with noise, and missing information inherent in expert-based pair wise comparisons and multi-criteria input variables.

More recently, MCDM-Machine Learning (ML) frameworks have emerged to incorporate predictive capabilities by combining fuzzy weighting, and machine learning to simultaneously manage uncertainty, subjectivity, and forecasting challenges in site selection of renewable energy. However, these hybrid approaches predominantly rely on subjective expert judgments for weight assignment and criterion evaluation that lacks the capacity to incorporate objective performance benchmarking in the decision-making process. To date, no research that incorporates to handle subjective, objective, predictive machine learning capabilities in decision making process of suitable location in wind farm site that can handle both uncertainty and generalization.

Despite Ethiopia’s Amhara Region offering substantial wind energy potential, existing site selection frameworks remain limited by static AHP and statistical methodologies that cannot generalize beyond initial datasets^21^. It struggles with noise and missing data in expert assessments and fail to provide predictive capabilities for unsampled locations that necessitates an integrated FAHP-DEA-ML approach to combines fuzzy weighting, objective efficiency screening, and machine learning generalization to deliver actionable wind farm corridors in data-scarce environments.

The paper presents three stage integrated framework (FAHP-DEA-ML) for wind farm strategic site selection in Ethiopia’s Amhara region. Thus, the objective of the paper is to prioritize the most promising wind development zones of Amhara region among the FAHP-identified suitable areas by applying DEA by treating each zone as a decision-making unit and assess its relative efficiency under multiple input–output indicators. A DEA prioritized fuzzy-weighted spatial suitability map generated by FAHP have been applied to ML model to predict and validate long-term wind potential and site performance for final site selection.

Additionally, the paper aimed to construct and train a machine learning model using the features and labels derived from DEA-identified “most suitable” areas, with the aim of learning a spatial suitability function that can be generalized to unsampled locations. Finally, the work delineates wind farm corridor and estimate potential area within the predicted priority zones for deployment and further techno economic studies.

The major contributions of this paper are as follows:

(i) it presents the first integrated framework combining FAHP, DEA, and Machine Learning for strategic wind farm site selection in data-scarce regions, specifically applied to Ethiopia’s Amhara Region. The framework has also been validated by planned projects and different current literatures.
(ii) it fills a critical methodological gap by developing an intelligent framework that overcomes three fundamental limitations of existing approaches such as static nature, limited generalization, data quality issues.
(iii) it also advances wind energy planning theory by demonstrating subjective weighting, objective efficiency analysis, and predictive modelling to create a unified strategic decision-support system applicable to data-scarce regions globally, supporting UN SDG7 and sustainable energy transitions.

This paper is then organized as: section “Related works and gap identification” reviews related literatures of DEA-MCDM and ML-MCDM techniques for renewable energy site suitability to identify possible research silos, section “Mathematical foundations on FAHP and DEA” presents mathematical foundations for DEA and FAHP, section “Integrated FAHP-DEA-ML methodology” illustrates methodology that integrates three different techniques such as DEA, FAHP and ML, section “Result analysis” reports result analysis, section “Discussion” discussions on the findings and finally conclusion and future work are placed in section “Conclusion and recommendations”.

## Related works and gap identification

A comprehensive review of prior research is essential to understand the current state of integrated assessment methodologies for strategic wind farm siting and to identify opportunities for methodological advancement in the field. This section reviews the literature in framework of DEA_MCDM with their integration with machine learning approaches and possible research gap.

### Related previous works

While traditional DEA-MCDM frameworks have demonstrated effectiveness in renewable energy site selection processes, their reliance on predetermined weights and crisp datasets limits their capacity to address uncertainties inherent in expert judgments. This challenge has driven researchers to explore hybrid methodologies that combine DEA’s efficiency measurement capabilities with advanced decision-making tools capable of handling vagueness and spatial complexity. On the other hand, machine learning has been integrated with advanced decision-making tools to add predictive capability on the spatial patterns. The evolution of these integrated approaches can be traced through three progressive waves of research: early DEA-MCDM applications in PV and wind site selection, fuzzy extensions for linguistic uncertainty management, and recent ML-enhanced MCDM frameworks that leverage spatial intelligence.

The integration of DEA with MCDM methods has emerged as a robust framework to evaluate and select sites for renewable energy projects in order to address the multifaceted challenges of sustainability, resource efficiency, and environmental impact. DEA, a non-parametric technique for measuring relative efficiency of decision-making units (DMUs), excels in handling multiple inputs and outputs without predefined functional forms^19^. DEA is particularly suitable for renewable energy assessments where factors like terrain, grid proximity, and energy yield vary significantly^19–23^.

Early applications of DEA in renewable energy focused on photovoltaic (PV) site selection with emphasis on sustainability criteria. Authors in^19^ proposed a novel non-radial DEA algorithm combined with clustering to identify optimal PV locations in Iran. Their single- and multi-period models incorporated inputs such as solar irradiation and land availability, alongside outputs like energy yield and environmental benefits. By evaluating 31 provinces, the study identified six high-potential sites to demonstrate alignment with Iran’s PV potential maps. The clustering step resolved discrepancies between radial and non-radial efficiencies that reveals multi-period analysis better captures temporal sustainability dynamics, such as resource depletion over time.

Building on this, hybrid DEA-MCDM models have gained attraction for wind and solar farm site selection in Asia-Pacific contexts. Authors in^20^ developed a two-stage DEA-analytic hierarchy process (AHP) methodology for wind power plant sites in Vietnam. In the first stage, six DEA variants screened 12 coastal provinces using five inputs and output such as frequency of disaster, topographic area, wind speed, grid distance and population. The result underscores the method’s efficacy in filtering alternatives amid Vietnam’s 512 GW wind potential. The second-stage AHP weighted 20 sub-criteria across five main factors (technical, economic, social, environmental, policy), with “support mechanisms” as the dominant influencer. Sensitivity analysis confirmed robustness by reducing computational complexity by 40% compared to standalone MCDM.

Similar hybrid frameworks have been applied to solar PV in seismically active regions. In^22^ DEA-AHP for 20 Taiwanese counties using meteorological inputs (temperature, humidity) and outputs (sunshine hours, insolation) to filter efficient sites has been employed. Three counties ranked top, driven by southern Taiwan’s irradiation greater than 145 W/m². AHP then prioritized via five criteria such as site characteristics, technical, economic, social, environmental resulted in electric power transmission cost and electricity consumption demand 0.086 as key sub-criteria. The study addressed land scarcity of Taiwan’s with 650 persons/km² density and projecting 20 GW capacities by 2025. Compared to GIS-based methods, this approach integrated socio-economic factors, improving decision transparency by 25%.

To handle linguistic uncertainties in qualitative criteria, grey-based extensions of MCDM have been integrated with DEA for solar site selection. Authors in^24^ proposed a two-stage DEA-Grey MCDM (G-AHP and G-TOPSIS) framework for PV power plants in Vietnam. DEA screened high-efficiency locations from 63 provinces using quantitative inputs (solar irradiation, land area) and outputs (energy production, CO₂ reduction). The top sites were then ranked via G-AHP and G-TOPSIS in order to incorporate grey numbers for expert judgments on 16 sub-criteria. The top ranked sites were aligned with Vietnam’s 20 GW solar target by 2030. This grey approach mitigated vagueness in policy-driven factors outperforming crisp AHP by 18% in robustness tests and emphasized cost savings through early-stage filtering.

Recent developments have extended DEA to fuzzy environments and broader renewable energy resource prioritization, explicitly addressing uncertainties inherent in expert judgments. In^23^ integrated DEA with fuzzy AHP (FAHP) to rank seven RES such as solar, hydro, biomass, onshore/offshore wind, wave, geothermal in India. Using a modified Ratio model to transform undesirable outputs like emissions, DEA computed initial efficiencies under four parameters and FAHP weighted parameters that yield overall scores of offshore wind while geothermal lagged. This framework filled gaps in Indian studies by including underrepresented sources like wave energy, aligning with net-zero goals by 2070.

Most recent advancements have shifted toward integrating machine learning (ML) with GIS and MCDM for wind farm site selection to catch predictive capabilities for handling spatial complexities. Authors in^25^ and^26^ developed a GIS-based ML approach using support vector machine (SVM) and random forest (RF) models for wind farm siting in Ekiti State, Nigeria and Philippine respectively. SVM processed spatial data to classify high-potential sites to outperform RF model in accuracy and efficiency in the case of Philippine whereas RF demonstrated superior predictive accuracy, identifying optimal upland sites for Nigeria. Similarly, in^29^ applied ensemble ML (RF, KNN, SVM, NB) with GIS in Türkiye, processing wind speed, elevation, and slope for suitability classification across diverse terrains across Black/Aegean/Mediterranean coasts. the intersected ensemble map pinpointed coastal hotspots that aligned with 20 GW onshore targets amid economic and population growth with accuracies reaching 95% in SVM. Hybrid cluster-ML-MCDM approaches have further refined wind site optimization in diverse geographies. Authors in^28^ introduced a cluster-based hybrid ML-MCDM framework for Iran using K-means clustering on 12 criteria to segment 31 provinces then followed by RF and AHP for prioritization. This study indicated ML’s role in enhancing traditional GIS analyses by reducing human bias and improving site validation. Authors^46^ in (2025) proposed a novel GIS-SF-AHP-WASPAS framework for onshore wind farm site selection in Morocco that integrated Spherical fuzzy AHP for standardization, WASPAS (Weighted Aggregated Sum Product Assessment) for ranking and machine learning for validation. The work is validated by existing farms and ML models (XGBoost R²=0.9982) and outperforms AHP/FAHP baselines. Machine learning models are objectively applied to validate the framework rather than prediction.

For enhanced interpretability, explainable AI (XAI) techniques have been incorporated into ML-driven site selection. Authors in^27^ introduced an ML framework using seven algorithms for automated wind farm siting in Balıkesir Province, Turkey, which hosts 13% of the nation’s wind capacity. SHAP explanations identified wind speed, transmission line proximity, protected zones, and elevation as top influencers. This XAI integration addressed MCDM’s subjectivity, revealing untapped high-potential areas for future 300 GW onshore expansions in Turkey. Authors of^30^ pioneered XAI-driven suitability mapping using RF/SVM/MLP on 13 factors and SHAP confirmed technical-economic dominance to map high wind and solar suitability. The previously ML based studies are summarized in terms of methods, models accuracy and validation technique applied in Table 1.

**Table 1:** Summary of literature on machine learning based suitability assessment.

Authors	Models applied	Methods	Accuracy of ML	Validation
^25^	SVM, RF	GIS-based machine learning, predictive modeling	AUC: SVM 0.75, RF 0.8	Area under the curve (AUC)
^28^	K-Means + + clustering	Machine learning integrated with MCDM and BORDA	Correlations > 0.9	Pearson and Spearman correlations
^30^	RF, SVM, Multi-layer Perceptron (MLP)	Explainable AI (XAI) with SHAP, GIS- based spatial suitability mapping	Overall: RF 90% (wind), 89% (solar); Kappa: 0.79 (wind), 0.78 (solar)	Overall accuracy, Kappa coefficient and AUC
^29^	RF, K-Nearest Neighbor, SVM, Naive Bayes	GIS with ensemble machine learning, classification of suitable/unsuitable sites based on wind speed, elevation, slope	K-Nearest Neighbor 93.022%, Random Forest 93.018%, Support Vector Machines 95.095%, Naive Bayes 89.553%	Accuracy metrics
^27^	XGBoost, Light Gradient Boosting, RF, Histogram-based Gradient Boosting, Logistic Regression, Naive Bayes	Explainable ML- based framework with feature selection, statistical tests, SHapley Additive exPlanations (SHAP)	XGBoost-0.9607, LightGBM- 0.9580, RF- 0.9518, HGB- 0.8946, LR- 0.8856, NB 0.8456	McNemar’s tests, agreement 97% with existing sites
^26^	SVM, RF	Integrated machine learning and MCDM, GIS-based analysis using datasets (wind speed, slope, roads, urban areas, protected areas, etc.)	AUC: SVM 0.75, RF 0.78	Area under the curve (AUC)
^46^	XGBoost, MLR	Machine learning models for validation of the Spherical Fuzzy-Analytic Hierarchy Process- (WASPAS) suitability map	XGBoost outperform MLR with (R^2^ = 0.9982, RMSE = 0.0043, MAE = 0.0016, MAPE = 0.5486)	With the existing project

### Gap identification

This paper identifies a critical methodological gap in wind farm site selection frameworks for data-scarce regions. Existing approaches using FAHP and DEA remain static and limited to ranking outcomes without enabling predictive decision support.

Despite extensive applications of FAHP and DEA in renewable energy site selection, existing hybrid models cannot generalize beyond the initial dataset and struggle with noise and missing information inherent in expert-based assessments and multi-criteria input variables. Thus, a critical research gap persists that no prior research has leveraged machine learning to learn from FAHP–DEA results to enhance forecasting accuracy, improve data quality, reduce information loss, and support dynamic planning as environmental, socio-economic, and technical conditions evolve. There exists methodological gap for developing an intelligent framework that integrates FAHP and DEA with machine learning to provide not only optimal site selection but also data-driven and continuously improving predictions for strategic site selection of wind power plant site for data scarce region.

While DEA provides objective efficiency baselines^19^, FAHP hybrids mitigate subjectivity^20–24^, and involvement of ML enable predictive mapping^25–30^. But gaps persist in unified frameworks handling vagueness via fuzzy logic, objectivity via DEA, and foresight via ML for dynamic wind farm suitability formulation at the same time. The proposed FAHP-DEA-ML integration addresses this by fuzzy-weighting criteria for uncertainty, DEA-scoring for efficiency, and ML-forecasting that offers robust site selection for wind farms in emerging markets to bridges literature silos toward sustainable energy transitions that align with UN SDG7.

## Mathematical foundations on FAHP and DEA

This section presents two fundamental mathematical foundations used in methodologies employed for site suitability selection of wind power plant: Fuzzy Analytic Hierarchy Process for criteria weighting and Data Envelopment Analysis for efficiency evaluation.

### Fuzzy analytic hierarchy process

The Analytic Hierarchy Process (AHP), introduced by Saaty, provides a structured methodology for solving complex multi-criteria decision-making (MCDM) problems^8–10^. However, classical AHP cannot represent the vagueness and ambiguity that characterize human judgment^10–18^. To address this, fuzzy set theory was incorporated into AHP, leading to the development of various Fuzzy AHP (FAHP) models by Van Laarhoven, Pedrycz, Buckley, and Chang^13,16^.

This study adopts the Buckley Fuzzy AHP (Geometric Mean FAHP) because it is computationally stable, free from rank reversal issues, and commonly used in GIS-based energy planning and wind farm suitability analysis^16^.

The FAHP steps followed in this work are outlined below.

Step 1: Construction of Fuzzy Pairwise Comparison Matrix.

Experts evaluate the relative importance of criteria using linguistic terms, which are converted into triangular fuzzy numbers (TFNs). The fuzzy scale used in this study is shown in Table 2 to construct pairwise matrix^13^. Five experts from Ethiopian Electric Utility, Adama Wind Farm, and Bahir Dar University participated in the elicitation process.

**Table 2:** Fuzzy conversion scale.

Linguistic term	Crisp value	TFN $\left( {l,m,u} \right)$	Reciprocal TFN
Equally important	1	$\left( {1,1,1} \right)$	$\left( {1,1,1} \right)$
Moderately important	3	$\left( {2,3,4} \right)$	$\left( {1/4,1/3,1/2} \right)$
Strongly important	5	$\left( {4,5,6} \right)$	$\left( {1/6,1/5,1/4} \right)$
Very strongly important	7	$\left( {6,7,8} \right)$	$\left( {1/8,1/7,1/6} \right)$
Extremely important	9	$\left( {8,9,10} \right)$	$\left( {1/10,1/9,1/8} \right)$
Intermediate values	$2,4,6,8$	Derived proportionally	

Step 2: Fuzzy membership functions

Triangular membership functions (TMFs) are the most widely used in FAHP applications due to their computational simplicity, consisting of three parameters (lower bound, peak, upper bound) that enable fast processing and straightforward interpretation of linguistic variables such as “low,” “medium,” and “high,” whereas Gaussian membership functions (GMFs) provide smoother, bell-shaped curves defined by mean and standard deviation that better represent natural uncertainty distributions but require more complex calculations^14,15^.

A triangular fuzzy number ( $l,m,u$ ) is represented using the membership function^13–16^:

1 $$\begin{array}{*{20}{c}} {\mu \left( x \right)=\left\{ {\begin{array}{*{20}{l}} {0,}&{x<l} \\ {\frac{{x - l}}{{m - l}},}&{l \leqslant x \leqslant m} \\ {\frac{{u - x}}{{u - m}},}&{m<x \leqslant u} \\ {0,}&{x>u} \end{array}} \right.~~} \end{array}$$

Step 3: Fuzzy Weight Calculation (Buckley’s Geometric Mean Method).

For each criterion *i*, the fuzzy geometric mean is computed as^13^:

2 $$\begin{array}{*{20}c} {G_{i} = \left( {\mathop \prod \limits_{{j = 1}}^{n} a_{{ij}} } \right)^{{1/n}} ~~} \\ \end{array}$$

where ${a_{ij}}$ is a triangular fuzzy number.

Normalized fuzzy weights are obtained as^14^:

3 $$\begin{array}{*{20}c} {w_{i} = \frac{{G_{i} }}{{\mathop \sum \nolimits_{{k = 1}}^{n} G_{k} }}} \\ \end{array}$$

This yields the fuzzy priority vector.

Step 4: Defuzzification and Final Normalization.

The fuzzy weights are converted into crisp values using the centroid (gravity) method^13–15,18^:

4 $$\begin{array}{*{20}{c}} {A{w_i}=\frac{{{l_i}+{m_i}+{u_i}}}{3}~} \end{array}$$

The final normalized crisp weights are:

5 $$\begin{array}{*{20}c} {w_{i}^{{crisp}} = \frac{{Aw_{i} }}{{\mathop \sum \nolimits_{{k = 1}}^{n} Aw_{k} }}~} \\ \end{array}$$

Step 5: Consistency evaluation.

It is very important to perform AHP consistency test after defuzzifying the fuzzy matrix for acceptable value. The Consistency Ratio (CR) is defined as^7,8^:

6 $$\begin{array}{*{20}{c}} {CR=\frac{{CI}}{{RI}}~~} \end{array}$$

where the Consistency Index (CI) is^8^:

7 $$\begin{array}{*{20}{c}} {CI=\frac{{{\lambda _{{\mathrm{max}}}} - n}}{{n - 1}}~} \end{array}$$

and the principal eigenvalue is approximated by^13^:

8 $$\begin{array}{*{20}c} {\lambda _{{{\mathrm{max}}}} = \frac{1}{n}\mathop \sum \limits_{{i = 1}}^{n} \frac{{(Aw)_{i} }}{{w_{i}^{{crisp}} }}~} \\ \end{array}$$

Consistency is acceptable when $CR<0.1$^8^.

RI- is the average CI of random generated pairwise matrices of the same order n and the common value are given in Table 3^8,17^. The consistency rate should be less than 10%. If it is higher or equal to 10%, the judgement in the matrix should be revised^6^.

**Table 3:** Random Index value for matrices of different size.

*n*	2	3	4	5	6	7	8	9
RI	0.00	0.52	0.89	1.11	1.25	1.32	1.41	1.45

### Data envelopment analysis (DEA)

To objectively measure the relative efficiency of potential wind farm sites (DMUs), this study employs three complementary and super-efficiency variant under variable return to scale (VRS) DEA models, namely, the Charnes-Cooper-Rhodes (CCR) under Constant Returns to Scale (CRS) for overall technical efficiency, the Banker-Charnes-Cooper (BCC) under VRS for pure technical efficiency, the input-oriented Slacks-Based Measure (SBM) under VRS for non-radial slack minimization and Super-Efficiency SBM Model variant under VRS to handle frontier bottleneck. These models are adapted from hybrid DEA frameworks in renewable site selection^19–24^. Inputs include distance to grid (km) and slope (degrees), Temperature (°C) while outputs comprise wind speed (m/s), coverage area (km^2^), and population that aligned with sustainability criteria in^20,22^. CCR assumes CRS for scale-invariant assessments, BCC incorporates VRS via convexity for heterogeneous terrains, SBM captures disproportionate inefficiencies, and Super-Efficiency SBM complements standard SBM by providing discrimination among efficient units. For undesirable outputs (if extended, e.g., emissions from^23^, apply the modified Ratio model^22^: Transform via $y_{r}^{\prime }=\frac{1}{{{y_2}}}$​ before evaluation. Efficient sites should be scores $\geqslant$ 0.95 and the top site among efficient sites should advance to ML stages. Consider the given initial structure decision variables and efficiency scores given in Table 4 for DEA model analysis.

**Table 4:** DEA Model structure: decision variables and efficiency metrics.

Given	Decision Variables and scores
Given for n DMUs, each consumes ‘m’ input and produce ‘s’ output are	**SBM Model Variables**
Inputs: $\:{x}_{ij}\ge\:0,\:i=1,\dots\:\dots\:\dots\:.m$	ρ: Slack-based efficiency score (objective function to minimize)
Outputs:$\:{y}_{rj}\ge\:0,\:r=1,\dots\:\dots\:\dots\:.s$	*λ*: Vector of intensity variables for constructing reference DMUs
DMUs: j = 1,……….n with DMUo under evaluation	*x*_*io*_: Input i value for DMUo (denominator in input slack ratio)
**CCR Multiplier Model Variables**	*y*_*ro*_: Output r value for DMUo (denominator in output slack ratio)
***v***: Vector of input weights assigned by the model	$\:\delta\:$= efficiency scores
***u***: Vector of output weights assigned by the model	$\:{s}_{i}^{-}\ge\:0$ –input slacks
***x***_***o***_: Input vector for DMUo	$\:{s}_{r}^{+}\ge\:0$ –output slacks
***uy***_***o***_: Weighted output score (objective function to maximize)	u-weight-input vector
**BCC Model Variables**	v-weight output vector
*θ*: Input reduction factor (efficiency score), where θ ≤ 1θ ≤ 1	**Efficiency Interpretation**
***λ***_***j***_: Intensity variables (weights) for DMUj in constructing the efficient frontier	**CCR Model**: Efficient if optimal value = 1 (Constant Returns to Scale)
***x***_***io***_: Value of input i for DMUo	**BCC Model**: Efficient if θ∗ = 1 (Variable Returns to Scale)
***y***_***ro***_: Value of output r for DMUo	**SBM Model**: Efficient if ρ∗ = 1with zero slacks

**Input-oriented CCR under Constant Returns to Scale**: DMU_0_ must be evaluated with the following Linear programming formulation in multiplier form^20^.

9 $$\begin{array}{*{20}{c}} {max\mathop \sum \limits_{r} {u_r}{y_{r0}}~} \end{array}$$

Subject to

$$\begin{array}{*{20}c} {\mathop \sum \limits_{i} v_{i} x_{{i0}} = 1~~} \\ \end{array}$$

$$\begin{array}{*{20}c} {\mathop \sum \limits_{r} u_{r} y_{{rj}} - \mathop \sum \limits_{i} v_{i} x_{{ij}} \le 1,~\forall j~} \\ \end{array}$$

$$\begin{array}{*{20}{c}} {{u_r} \geqslant ~{v_i} \geqslant \varepsilon >0~~} \end{array}$$

**BCC envelop model**: The envelopment form minimizes input reduction$~\theta$, projecting onto the convex hull ( $\sum ~{\lambda _j}=1$)^20,22,23^.

10 $$\begin{array}{*{20}l} {\mathop {{\mathrm{min}}}\limits_{{\theta ,\lambda }} } \hfill & \theta \hfill \\ {{\mathrm{~subjected~to~~}}} \hfill & {\theta x_{{io}} \ge \mathop \sum \limits_{{j = 1}}^{n} \lambda _{j} x_{{ij}} ,{\mathrm{~~for~all~}}i = 1, \ldots ,m} \hfill \\ {} \hfill & {y_{{ro}} \le \mathop \sum \limits_{{j = 1}}^{n} \lambda _{j} y_{{rj}} ,{\mathrm{~}}r = 1, \ldots ,s} \hfill \\ {~~~~~~~~~~~~~~~~~~~~} \hfill & {\mathop \sum \limits_{{j = 1}}^{n} \lambda _{j} = 1} \hfill \\ {} \hfill & {1 \ge \theta \ge 0{\mathrm{~~}},{\mathrm{~}}\lambda \ge 0} \hfill \\ \end{array} ~$$

Efficient if $${\theta ^{\mathrm{*}}}=1.$$

In multiplier form, Input-oriented BCC under Variable Returns to Scale can be expressed as follows:

It adds a free variable to capture variable returns to scale (increasing or decreasing)^19,20,22,24^

11 $${\mathrm{max}}\mathop \sum \limits_{r} u_{r} y_{{r0}} + u_{0}$$

Subject to

$${~\mathop \sum \limits_{i} v_{i} x_{{i0}} = 1~~}$$

$$u_{r} \ge 0,{\mathrm{~}}v_{i} \ge 0,{\mathrm{~}}v{\mathrm{X}} - u{\mathrm{Y}} - u01 \ge 0,u_{0} ~~ - {\mathrm{free}}$$

Notes.

- u_0_ determines increasing / decreasing / constant returns to scale and is the free variable representing the VRS condition.
- If u_0_ = 0, the model reduces to CCR.

Input-oriented SBM: Minimizes average slack-based inefficiency, $\:\rho\:$, handling nonradial slacks assuming equal weights^24^.

12 $$\begin{array}{*{20}{r}} {\mathop {{\mathrm{min}}}\limits_{{\lambda ,{s^ - },{s^+}}} }&{\rho =\frac{{1 - \frac{1}{m}\mathop \sum \nolimits_{{i=1}}^{m} \frac{{s_{i}^{ - }}}{{{x_{io~~~}}}}}}{{1+\frac{1}{s}\mathop \sum \nolimits_{{r=1}}^{s} \frac{{s_{r}^{+}}}{{{y_{ro}}}}}}} \\ {{\mathrm{~s}}{\mathrm{.t}}{\mathrm{.~~}}{x_{io}}}&{~=\mathop \sum \limits_{{j=1}}^{n} {\lambda _j}{x_{ij}}+{s^ - }{\mathrm{~~}}\forall {\mathbf{i}}} \\ {{y_{ro}}}&{~=\mathop \sum \limits_{{j=1}}^{n} {\lambda _j}{y_{rj}} - s_{r}^{+}{\mathrm{~~~}}\forall {\mathbf{r}}} \\ {{\lambda _j}}&{~ \geqslant 0,{\mathrm{~}}{s^ - } \geqslant 0,{\mathrm{~}}{s^+} \geqslant 0} \end{array}$$

Efficient if ${\rho ^{\mathrm{*}}}=1$ (zero slacks). Unit-invariant.

The Eq. (12) can be transformed to linear for using Charnes–Cooper transformation using^19,24^

13 $$\delta =\frac{1}{{1+\frac{1}{s}\mathop \sum \nolimits_{1}^{r} \frac{{s_{r}^{+}}}{{{y_{ro}}}}}}$$

Then the linear model for linear program becomes

14 $$\mathop {{\mathrm{min}}}\limits_{{\delta ,\lambda ,{s^ - },{s^+}}} \delta - \frac{\delta }{m}\mathop \sum \limits_{{i=1}}^{m} \frac{{s_{i}^{ - }}}{{{x_{io}}}}$$

$${\lambda _j} \geqslant 0,~{s^ - } \geqslant 0,~{s^+} \geqslant 0,~\delta >0$$

## Integrated FAHP-DEA-ML methodology

This section illustrates study area, methodological framework description, data sources and implementation procedures of each step for the proposed FAHP-DEA-ML method.

### Study area

**Fig. 1:**
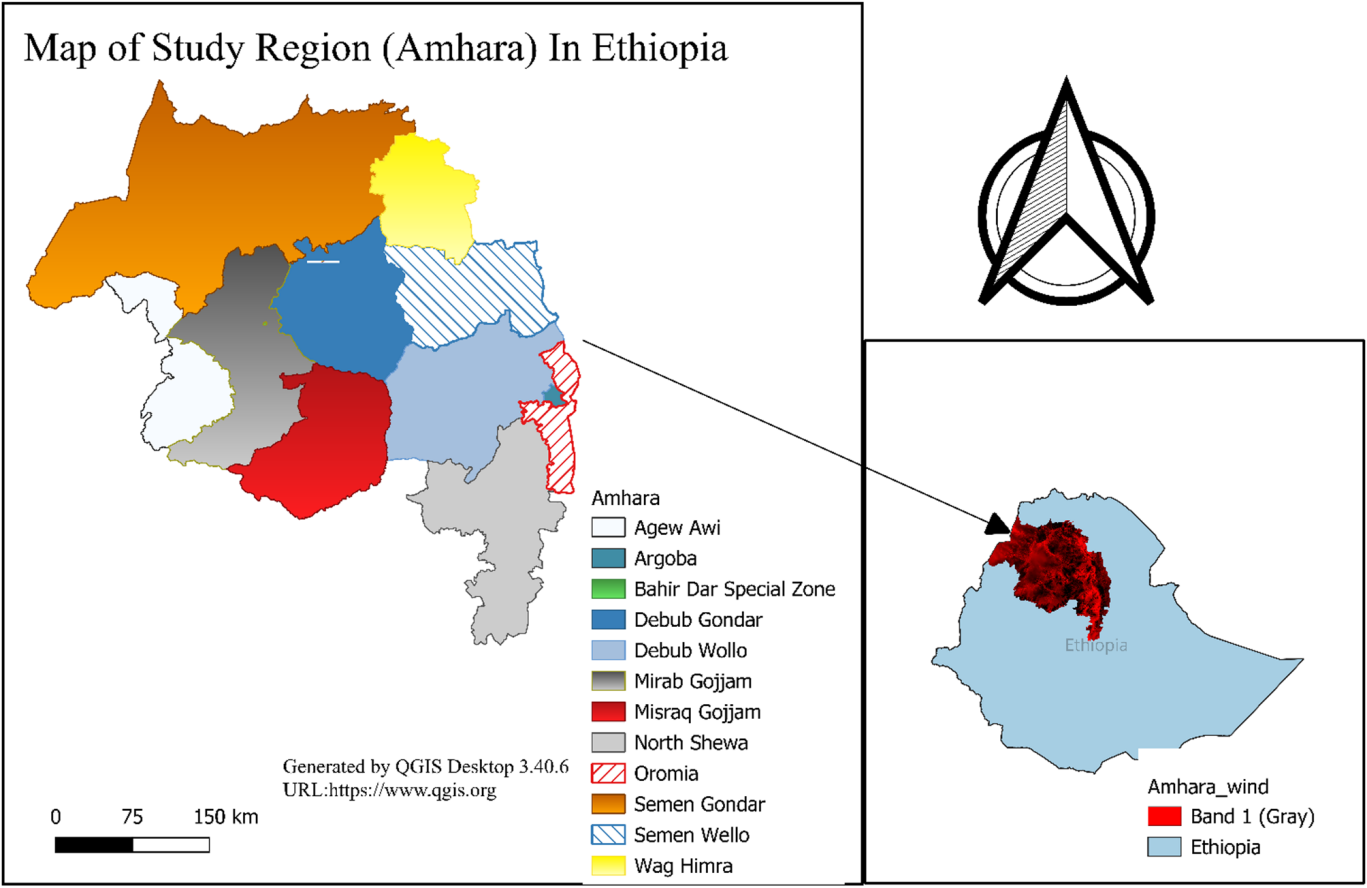
Location and administration map of Amhara region in Ethiopia.

The research took place in Ethiopia’s Amhara region, situated in the northern and north-eastern areas of the country, which is divided into twelve administrative zones as depicted in Fig. 1. Its varied landscape spans from Ethiopia’s tallest peak, Ras Dejen at 4,620 m, down to lowlands of 500 m above sea level, offering distinct prospects for various renewable energy types. The region’s hills, mountains, and valleys boost wind patterns that render it ideal for wind energy initiatives. Several investigations show favorable wind regimes in the area, with speeds surpassing 6.8 m/s^40^. Global Wind Atlas data reveals strong potential, with average power density reaching 218 W/m² at 100 m hub height^35^. This equates to an average wind speed of 6 m/s at the given height to align with thresholds for viable modern wind turbine operations.

### Methodological workflow

**Fig. 2:**
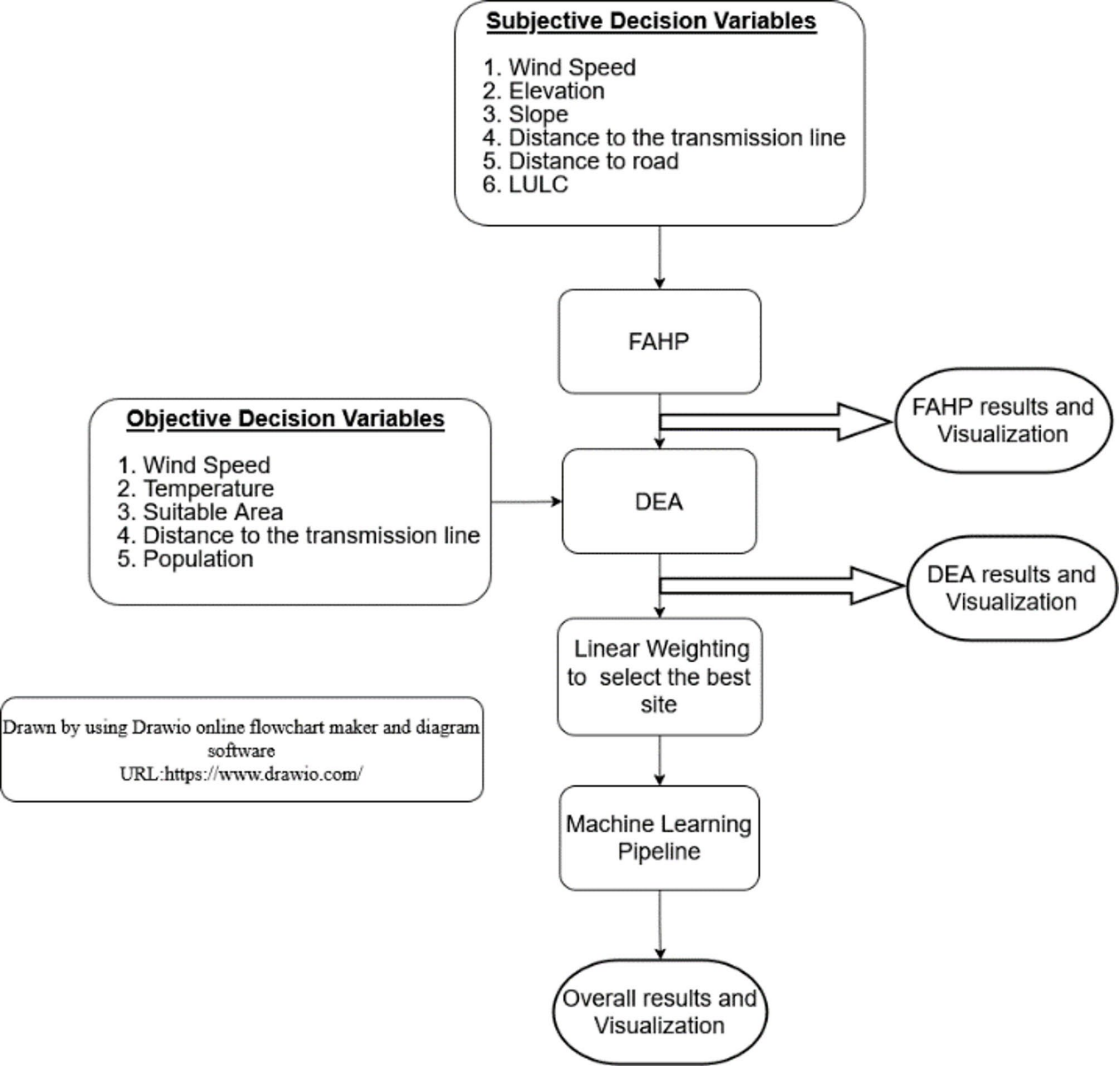
Integrated FAHP–DEA–ML framework for wind farm site selection.

This study employs a three-stage analytical framework integrating FAHP, DEA, and ML to identify, rank, and evaluate optimal energy infrastructure sites in the Amhara Region as shown in Fig. 2. Schematic overview of the proposed workflow showing how subjective and objective decision variables are processed via FAHP and DEA respectively, combined through linear weighting to identify the best site, and then passed to a machine learning pipeline for overall site selection results and visualization. First, FAHP is applied to incorporate expert judgement under uncertainty and generate a spatial suitability map by assigning fuzzy-weighted importance to technical, environmental, and socio-economic criteria. The resulting FAHP surface is used to extract candidate sites with high suitability scores. Second, these candidate locations are evaluated using input-oriented DEA (CCR, BCC, and SBM models) to determine their relative efficiency based on minimizing constraints such as distance to grid, slope of terrain, temperature and maximizing potential benefits like suitable area, average wind speed and population. DEA filters out inefficient alternatives and identifies the most promising sites through a non-parametric, performance-driven ranking. Finally, the top-ranked DEA-efficient sites are supplied to a Machine Learning pipeline, where models such as RF, SVM, and XGBoost are trained to predict site-level performance and generalize characteristics of high-quality locations. This integrated FAHP–DEA–ML workflow enables robust spatial screening, evidence-based site selection, and predictive evaluation to ensure both methodological rigor and practical relevance for energy planning.

### Determining the suitable location of wind power plant using FAHP

FAHP has been applied to select optimal locations of wind power plant in the Amhara Region of Ethiopia. Six sequential stages applied as systematically described in the following subsequent steps.

**Stage one-establishing the criteria**: Previous studies consistently pointed out the importance of integrating technical, economic, environmental, and social factors when selecting suitable sites^9–14^. Technical parameters such as wind speed, air density, and wind direction are essential for assessing energy potential^11–14^, while economic considerations that includes proximity to the power grid, major roads, and demand centres are significantly influence project feasibility^10,11^. Environmental aspects such as land-use/land-cover and distance from protected areas are critical to minimizing ecological impacts^9,14^, and social factors including distance from residential areas, social acceptance, and potential job creation contribute to project sustainability^12^. Together, these factors ensure that selected locations maximize energy generation while reducing financial and environmental costs^11–13^. To define the specific criteria for this study, a combined approach involving a review of existing literature and expert opinions collected through questionnaires was applied. This process resulted in six selected criteria: wind speed (C1), slope of terrain (C2), elevation (C3), proximity to high-voltage transmission lines (C4), distance from main roads (C5) and land use land cover (C6) as summarized in Table 5. Expert suggested criteria and four cited literatures have been applied to select six best criteria among 20 listed in the Table 4.

**Table 5:** Selected criteria from literature and experts.

Criteria	^11^	^12^	^10^	^9^	^13^	^14^	^15^	^26^	Experts	Proposed
Wind speed	✓	✓	✓	✓	✓	✓	✓	✓	✓	✓
Distance from road	✓	✓	✓	✓	✓	✓	✓	✓	✓	✓
Distance from transmission line	✓	✓	✓	✓	✓	✓	✓	✓	✓	✓
Slope of terrain	✓	✓	✓	✓	✓	✓	✓	o	✓	✓
Distance from residential area	✓	✓	✓	✓	o	✓	o	o	o	o
Land use land cover	o	✓	✓	✓	✓	o	o	o	✓	✓
Distance from Airport	✓	o	o	o	o	o	o	o	o	o
Land elevation	✓	o	o	o	✓	o	✓	✓	✓	✓
Distance to fault line	o	o	✓	✓	o	o	o	o	o	o
Distance to water bodies/ river	o	o	✓	✓	o	o	o	o	o	o
Distance to infrastructure	o	o	✓	✓	o	o	o	o	✓	o
Bird density	o	o	✓	o	o	✓	o	o	o	o
Aspect/orientation	o	o	✓	o	o	o	o	o	o	o
Ice zone	o	o	o	o	o	✓	o	o	o	o
Recreation sites	o	o	o	o	o	✓	o	o	o	o
Rocky land	o	o	o	o	o	✓	o	o	o	o
Forest land	o	o	o	o	o	✓	o	o	o	o
Roughness of terrain	o	o	o	o	o	✓	o	o	o	o
Geology	o	o	o	o	o	✓	o	✓	o	o
Forest zone	o	o	o	o	o	o	o	o	✓	o
Protected area	o	o	o	o	o	o	o	✓	✓	o

**Stage two-data sourcing**: The data required for evaluating wind farm siting criteria were obtained from various sources and in different formats, necessitating pre-processing before being used in the analysis. Table 6 summarizes the data sources and the types of datasets employed in this study.

**Table 6:** Data type and corresponding sources.

No	Criteria	Data type	Source	Link	Category of criteria
1	Wind speed	Raster	Global Wind Atlas	https://globalwindatlas.info	Technical criteria
2	Elevation (DEM)	Raster	U.S Geological Survey (USGS) downloaded using JavaScript on GEE console	Processed through GEE	Technical/ Environmental
3	Slope	Raster	Extracted from DEM	-	Environmental
4	Proximity to transmission line	Vector	NERGYDATA.INFO website (An Innovation of World Bank Group)	https://energydata.info/	Economic/ Technical
5	Proximity to main Road	Vector	Open street map export of humanitarian data exchange	https://data.humdata.org/	Economic
6	LULC	Vector	Agricultural Transformation Agency, Ethiopia	https://ati.gov.et/	Environmental/ Social

**Stage three- Buffering and suitability classification**: Buffering is applied to create exclusion zones around sensitive features to help balance technical, economic, environmental and social impacts, while suitability classification categorizes potential sites into levels such as Very Low, Low, Moderate, High, and Very High based on criteria including wind speed, slope, land use, and regulatory constraints, as shown in previous studies^12–15^. Together, these processes ensure the optimal placement of wind farms with minimal conflict. Table 7 summarizes the selected criteria, their recommended thresholds, corresponding suitability classes, and explanatory notes derived from expert opinions, and existing literature.

**Table 7:** Summary of short description for key criteria, suitability class and thresholds.

Ref.	Criteria	Threshold value	Suitability class	Description
^10–16,26^	Wind speed (C1)	< 3 and > 6.5 m/s	Very low (3–4 m/s), Low (4–5 m/s), Moderate (5–6 m/s), High (6–6.5 m/s), Very high (> 6.5 m/s)	Measures the average wind velocity (m/s) at the site. Higher wind speeds are critical for efficient energy generation. The wind speed data collected maximum speed in the region was 13.4 m/s. The minimum speed of 3 m/s is the cut-in speed of the turbine
^9–11,13,25^	Elevation (C2)	> 3000 m	Very low (2000–3000 m), Low (1500–2000 m), Moderate (1000–1500 m), High (750–1000), Very high (515–750 m)	The height above sea level (in meters). Moderate elevations are ideal to balance wind potential and construction feasibility. Air density decreases as with altitude and it is directly proportional to turbine output power
^12–14^	Slope (C3)	> 15%	Very low (12–15%), Low (9–12%), Moderate (6–9%), High (3–6%), Very high (< 3%)	Refers to the steepness of the terrain (in degrees or %). Flatter slopes are preferred for easier turbine installation and maintenance
^12,13^	Proximity to high voltage transmission (C4)	< 0.5 km	Very low (> 80 km), Low (40–80 km), Moderate (20–40 km), High (10–20 km), Very high (0.5–10 km)	Proximity to power grids (in km). Shorter distances reduce energy loss and connection costs
^13^,^14^	Proximity to main Road(C5)	< 0.5 km	Very low (> 50 km), Low (20–50 km), Moderate (10–20 km), High (5–10 km), Very high (0.5–5 km)	Proximity to existing roads (in km). Closer distances reduce transportation costs for equipment and maintenance
^13,17,25^	LULC(C6)	-	Very low (Aquatic and open water bodies), Low (Tree and crop cover), Moderate (Shrubs cover), High (grass and sparse land) and very high (Bare area)	Type of land (e.g., grass land, Shrubs, barren). Open, non- protected lands are preferred for minimal environmental impact

**Stage four-formation of pairwise comparison matrix**: Experts compared the criteria using linguistic terms converted into triangular fuzzy numbers (TFNs), after which a fuzzy pairwise comparison matrix was constructed, with each entry representing the relative importance of one criterion over another^13^. The final pairwise comparison matrix was generated by averaging the judgments obtained from domain experts and relevant literature, as presented in Table 7. Following the approach recommended in^10^ and^11^, this study interviewed five experts through questioner in the fields of Electrical and Mechanical Engineering from the Ethiopian Electric Utility head office, Adama Wind Farm, and Bahir Dar University.

**Table 8:** Pairwise comparison matrix for FAHP.

Criteria	C1	C2	C3	C4	C5	C6
C1	(1,1,1)	(5,6,7)	(4,5,6)	(3,4,5)	(2,3,4)	(4,5,6)
C2	(1/7,1/6,1/5)	(1,1,1)	(1,2,3)	(1/5,1/4,1/3)	(1/4,1/3,1/2)	(1/3,1/2,1)
C3	(1/6,1/5,1/4)	(1/3,1/2,1)	(1,1,1)	(1/4,1/3,1/2)	(1/3,1/2,1)	(2,3,4)
C4	(1/5,1/4,1/3)	(3,4,5)	(2,3,4)	(1,1,1)	(1,2,3)	(2,3,4)
C5	(1/4,1/3,1/2)	(2,3,4)	(1,2,3)	(1/3,1/2,1)	(1,1,1)	(1,2,3)
C6	(1/6,1/5,1/4)	(1,2,3)	(1/4,1/3,1/2)	(1/4,1/3,1/2)	(1/3,1/2,1)	(1,1,1)

**Ethical approval and informed consent**: All methods in this research were carried out in accordance with relevant guidelines and regulations involving human participants. The study was carried out non-invasive expert consultations conducted for the purpose of obtaining pairwise comparison judgments within the FAHP framework for strategic wind power plant siting in Ethiopia. No sensitive personal data were collected. Bahir Dar University has an established research ethics review structure. However, according to institutional regulations at the Institute of Technology of Bahir Dar University, formal ethical approval was not required for this type of non-clinical and voluntary expert-based study. Participation was voluntary and informed consent was obtained from all participants prior to their involvement. Confidentiality and anonymity of the participants were ensured throughout the research process.

**Fig. 3:**
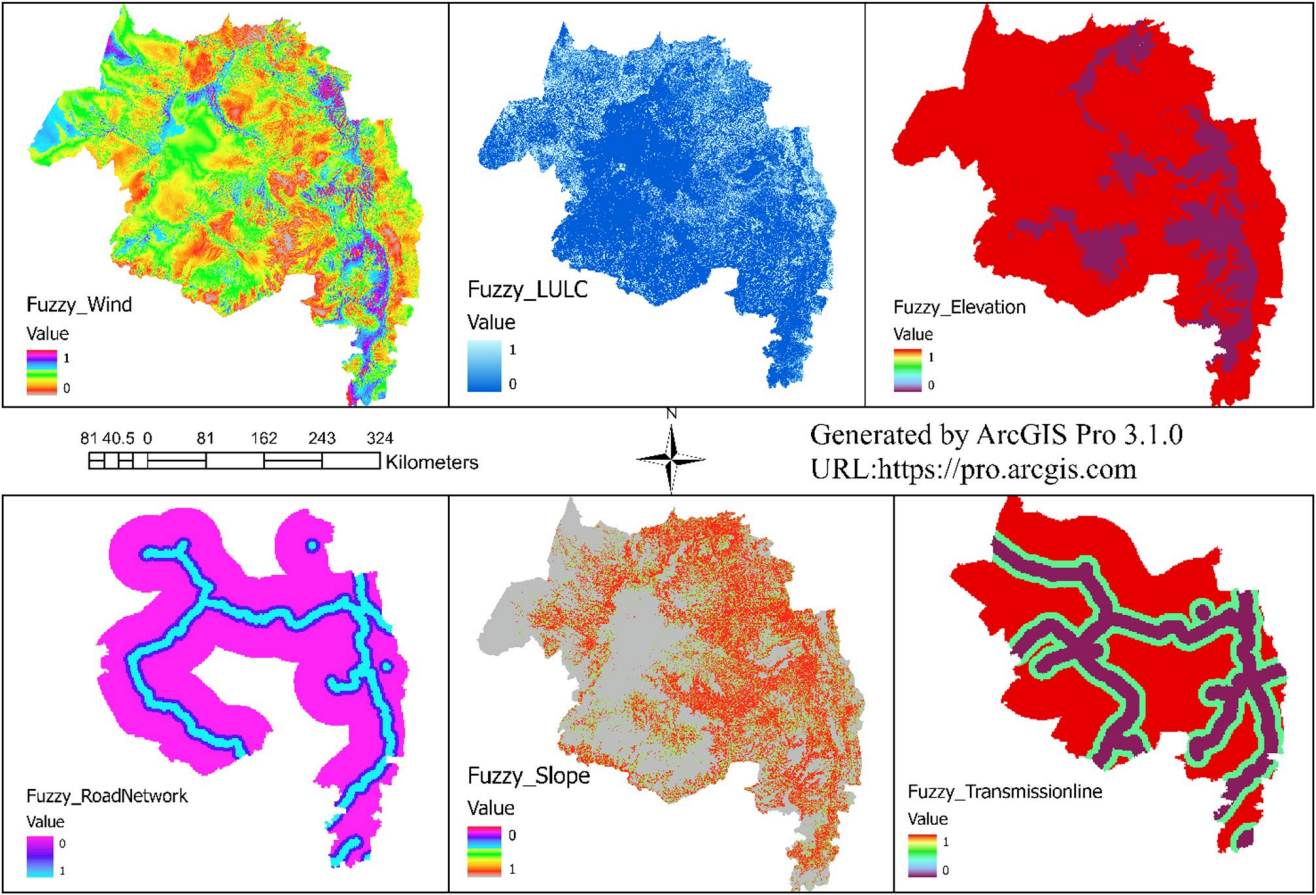
Fuzzified suitability maps of wind farm siting criteria.

**Stage five- FAHP-based fuzzy weighting and consistency checking framework**: In this stage, uncertainty in expert judgments is captured using triangular fuzzy numbers (TFNs), defined as (l, m, u)(l, m, u)(l, m, u) where l is the lower bound, m the modal value, and u is the upper bound, forming a fuzzy pairwise comparison matrix with reciprocal values computed as in Table 1^4,35^. The membership function of each TFN is modelled using the standard piecewise function shown in Eq. (1)^36^. Fuzzy weights are then derived using the geometric mean method, which is mathematically robust for FAHP applications^15,16,35^, and the resulting fuzzy weights are normalized using Eqs. (3) and (4). Finally, the fuzzy weights are defuzzified into crisp values using the centroid method—widely applied in wind farm site selection for its accuracy and stability^17,35^—and the results are normalized to obtain the final crisp weights for further consistency evaluation using Eq. (5). Fuzzy membership rasters and vector-based buffers for wind speed, land use/land cover, elevation, road network, slope, and transmission lines, showing normalized suitability values (0–1) across the study area is illustrated in Fig. 3.

**Stage six-** Apply the computed weight to rank spatial alternatives using weighted overlay technique in QGIS. Multi-criteria decision-making analysis using fuzzy analytical hierarchal Process has been done. In this work, spatial factor that influence wind energy development such as wind speed, slope, elevation, high voltage transmission network, main road network and land use land cover were integrated in to layers.

### Selection of best candidate using DEA

The suitable area in the region was evaluated using FAHP to pass the result through DEA pipeline for best performer selection. The suitable spaces in 12 different zones of the study area listed in Fig. 1 were considered as DMUs hereafter, and the area on square kilometres were identified in the FAHP results. The pipeline has four fundamental stages such as input/output identification, data collection, solve for CCR, BCC and SBM DEA models and identify the best performer to pass into the next step.

Based on the literature described in Table 8, a DEA specification for wind farm site selection can reasonably treat slope, distance to grid line, and temperature as inputs, because they increase construction difficulty, network extension cost, and affect turbine performance. The input can be described as “the lower the better”. Wind speed, suitable land area, and population in the service region can then be represented as outputs to reflect higher energy yield and greater socio-economic impact per unit of technical and economic effort. The output can also be described as “The higher the better”. For this work, based on previous studies and FAHP output of the first stage in this integrated framework, the proposed input and output are selected as shown in Table 9.

**Table 9:** Selection of input and output criteria.

Ref	DEA model type	Inputs used	Outputs used
^2^	CCR, BCC, SBM	Frequency of Natural Disasters, Average Land Cost, Connection Distance to Transmission Grid	Average Wind Blow, Population, Quantity of Proper Geological & Topographical Areas
^13^	FAHP- DEA	Land cost, Population density, Infrastructure distance, Land availability, Natural disaster risk, Environmental hazard indicators	Population, Suitable land area, Wind speed, Electricity consumption
^1^	Double-Frontier DEA	Human Development Index, Environmental burden, Geographic/technical constraints	Wind speed, Solar irradiation, Sustainability indicators, Hybrid energy output
^14^	Double-Frontier Network DEA (DF-NDEA)	Wind resource input, Solar radiation/irradiance, Environmental burden indicators	Wind energy output, Solar energy output, Combined hybrid performance, Environmental/social benefit outputs
Proposed	FAHP-DEA-ML	Temperature and distance to the grid	Suitable Land Area, Wind Speed and Population

Distance to grid, and average temperature indicators are modelled as **inputs** capturing infrastructure requirements, and atmospheric conditions affecting turbine performance. While population, average wind speed, and suitable area are modelled as **outputs** representing energy potential and socio-economic benefits of each candidate site and full description with corresponding sources are illustrated as follows.

Distance to the Grid^19^: Larger distances increase infrastructure requirements, line-extension costs, and transmission losses. From an input-oriented DEA perspective, shorter distances represent lower resource use, so efficient sites are those that deliver greater outputs while minimizing grid connection distance. This was digitalized from Google earth engine is shown in Fig. 3.

Average Temperature^49^: The temperature affects wind power plant performance primarily through its influence on air density and thermal conditions of turbine components. Colder air is denser, which exerts greater force on the turbine blades, increasing lift and, consequently, power output even at similar wind speeds. This is why wind turbines tend to produce more electricity during cold spells compared to warm weather. Temperature data of the site was collected from ECMWF/Copernicus for ERA5.

Population^13,20^: Locations closer to areas with higher population levels create greater socio-economic benefits that includes improved electrification and energy access. In DEA, population is considered an output because higher demand coverage increases the desirability and impact of the project. United Nation Humanitarian Data Exchange portal(https://data.humdata.org/) was used for this research.

Average Wind Speed^22,23^: Since the goal of wind farm siting is to maximize energy generation, wind speed is defined as an output in the DEA model, where efficient DMUs produce higher wind resource benefit given the same or lower input requirements. Global Wind Atlas (https://globalwindatlas.info) was the source of wind speed.

Suitable Area^22–24^: Suitable area refers to the amount of land within each candidate location that meets technical, environmental, social, economic and technical constraints for wind farm installation as depicted in FAHP section. Larger suitable areas offer more flexibility for turbine layout, potential for higher installed capacity, and economies of scale. In DEA, this is an output because locations with greater feasible land availability contribute more effectively to overall project potential. The suitable area was extracted from the result of FAHP using zonal Histogram in GIS software. The input and output parameters used in DEA model rationale is described in Table 10.

**Table 10:** DEA input-output variables, justifications, and literature alignment for wind farm site selection in Amhara Region.

Variable	Type	Justification	Literature alignment
Distance to grid	Input	Infrastructure cost/losses	^20^
Temperature	Input	Air density/performance	^49^
Wind speed	Output	Energy potential	^20,24^
Suitable area	Output	Capacity scale	^22–24^
Population	Output	Demand/socio-economic	^24^

According to the rule of described in^20^, the number of DMUs should be at least double higher than the total number of input and output variables in applying DEA. For this stage of DEA models, the authors considered five indicators for inputs and outputs as described above and 12 locations were nominated as DMUs: Awi/Agew, Bahir Dar Special Zone, North Gonder, South Wollo, Argoba Special Woreda, East Gojam, North Shewa, North Wollo, Oromia, South Gonder, Wag Himra and West Gojam.

### Machine learning models for wind farm site selection

This subsection demonstrated the machine learning approach employed to predict site suitability of wind power plant using FAHP and DEA efficiency resulted map as target variable. Six variables such as wind speed, slope of terrain, elevation, proximity to high-voltage transmission lines, distance from main roads and temperature were used as a predictor. This subsection also described the model selection process, training procedures, hyperparameter tuning, and validation methodology.

#### Model selection

From a comprehensive review of the literature on machine learning applications for wind farm site selection, the top-performing machine learning models based on reported metrics such as accuracy, precision, or kappa are Random Forest, Support Vector Machine and Extreme Gradient Boosting (XGBoost) for wind farm site selection as indicated in Table 11. Thus, three machine learning models have been tasted for performance for wind farm optimal suitability location selection for this work.

**Table 11:** Previous work performance metrics evaluation.

Authors (Year)	Best model	Key performance metric	Brief rationale
^25^	RF	AUC: 0.80	Outperformed SVM (AUC: 0.75) in predicting suitability using geo-factors like wind speed and slope; superior for handling spatial variability
^26^	SVM	Accuracy: ~92% (inferred from classification on Philippine datasets)	Primary model for classifying high-potential sites via topography and infrastructure; excelled in efficiency over traditional MCDM
^27^	XGBoost	Accuracy: 0.9607	Highest among 7 models (e.g., > LightGBM 0.9580, RF 0.9518); SHAP interpretability highlighted wind speed/ elevation as key, with 97% match
^28^	RF	Accuracy: 92%	Integrated with K-means clustering and AHP; best for prioritizing Tehran/Isfahan via 12 criteria, offering 15% scalability gain over non-ML hybrids
^29^	SVM	Accuracy: 95.09%	Top in ensemble (RF 93.02%, KNN 93.02%, NB 89.55%); intersected results reduced bias, mapping coastal hotspots for 20 GW potential
^30^	RF	Accuracy: 90% (wind); Kappa: 0.79	Outperformed SVM/MLP (AUC: 0.96 overall); SHAP validated technical-economic factors, mapping 26.84 M km^2^ global wind suitability

#### Pre-processing of input dataset

**Fig. 4:**
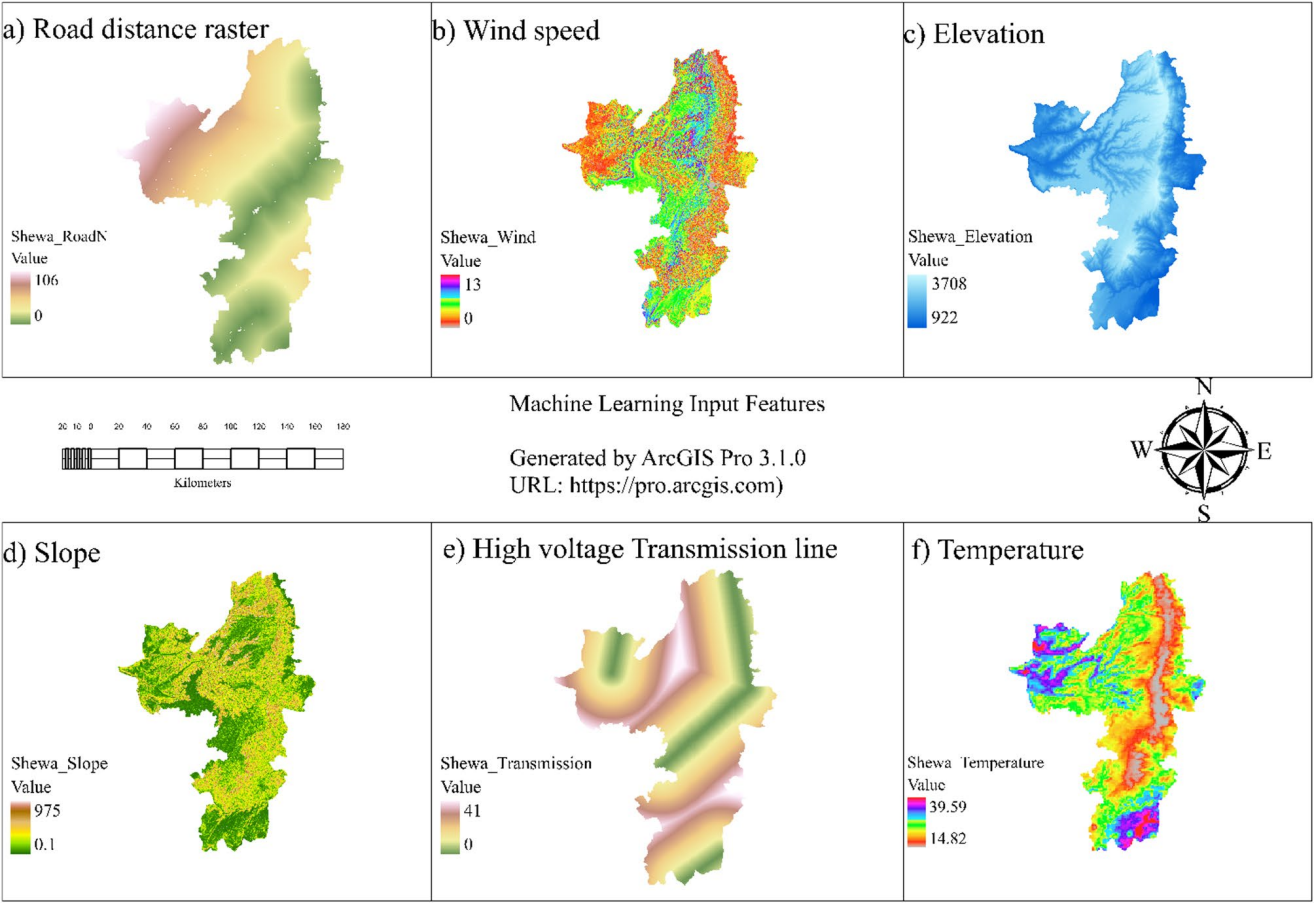
Spatially harmonized input feature layers for machine learning-based wind farm suitability prediction.

Preparation of the input feature dataset for the ML pipeline followed a rigorous geospatial pre-processing workflow to ensure spatial, radiometric, and geometric consistency across all predictors. All feature layers mapped in Fig. 4 such as distance to the road, distance to the grid, slope, elevation, wind speed and temperature were first projected into a common coordinate reference system (WGS 1984 UTM Zone 37 N) to eliminate spatial distortion and ensure uniform metric interpretation. Vector-based features were topologically cleaned, converted to continuous raster surfaces (e.g., Euclidean distance maps), and resampled to a standard spatial resolution aligned using a snap raster to guarantee pixel-level correspondence. All raster layers were clipped to the study boundary, normalized to reduce scale imbalance as shown in Fig. 4, and stacked into a single multi-band raster cube representing the ML feature space. This harmonized geospatial feature stack forms the input matrix for supervised learning, enabling the algorithm to correctly learn spatial patterns associated with optimal wind power plant sites, as derived from the FAHP–DEA hybrid suitability analysis.

#### Suitability mapping using machine learning

**Fig. 5:**
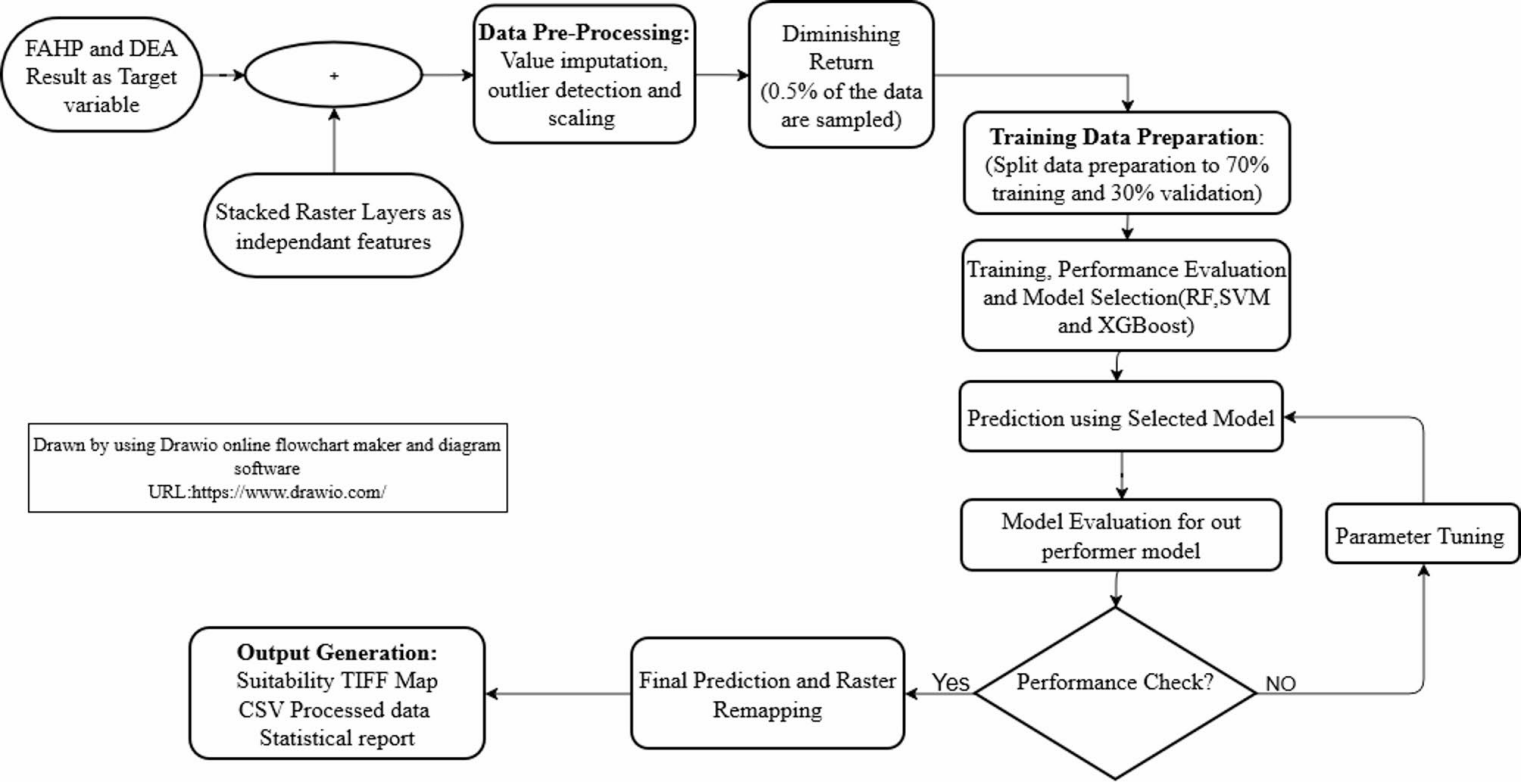
Machine-learning workflow for wind-farm suitability modelling using DEA–FAHP scores as targets and stacked raster layers as predictors.

The flow chart in Fig. 5 summarizes an integrated FAHP–DEA–ML workflow in which FAHP–DEA suitability scores are used as the target variable and stacked raster layers as predictors, followed by data pre-processing and diminishing-return sampling to prepare the training set. The data are then split into training and validation subsets, and models (Random Forest, SVM, and XGBoost) are trained, evaluated, and the best among them have been selected. The selected model was then iteratively tuned through successive parameter adjustments until the agreed performance criteria were satisfied, after which it was used to generate the final suitability predictions. These decision outputs were converted into spatial and tabular products through raster re-mapping and the creation of suitability GeoTIFF layers, CSV files, and associated statistical summaries.

#### Machine learning algorithms

Random Forest: Ensemble methods such as Random Forest are effective for analysing structured datasets. Among these methods, the bagging-based ensemble learning algorithmS haVe been widely applied in various research studies. This algorithm relies on the random selection of data samples and node characteristics parameters^40^. Random Forest Regression (RFR) is recognized for its strong generalization ability and fast training speed. It divides the input dataset into multiple subsets, builds a decision tree for each subset, and combines the outputs of these trees to generate the final prediction^41^. To enhance model performance, key hyper-parameters of the RFR model were tuned. Specifically, the number of estimators (n_estimators) was set to 500, and the maximum tree depth (max_depth) was limited to 12 to control tree growth and reduce the risk of overfitting. The final model parameters used in this work has been indicated in Table 12.

**Table 12:** Hyperparameter configurations and K-fold cross-validation settings.

Model	Key parameters	K-fold parameters
Random Forest	n_estimators = 500, max_depth = 12, random_state = 42 and default others	n_splits = 5, shuffle=True, r andom_state = 42
XGBoost	n_estimators = 500, max_depth = 6, learning_rate = 0.1, random_state = 42 a nd default others	
SVM	kernel=’rbf’, C = 10, gamma=’scale’, random_state = 42	

Support Vector Machine: Nonlinear Support Vector Regression (SVR) is the most widely used form of Support Vector Machine (SVM) for both classification and regression tasks. It constructs a line, plane, or hyper-plane in one-, two-, or multi-dimensional input space to classify data and predict continuous variables^26,29^. The objective of the SVR model is to find a function f(x) that deviates from the actual target values by no more than a specified margin epsilon (ε), while maintaining the flattest possible hyper-plane^26^. An advantage of a support vector machine is that the model being built is explicitly based on a subset of data points and support vectors that help interpret the model^26^. When selecting the type of kernel function that affects the result, the input dataset should be taken into account. The Support Vector Regression (SVR) model was optimized using a grid search strategy with cross-validation to identify the optimal hyper-parameters^40,41^. Because they could balance model complexity and predictive performance, the values of C = 10 and gamma = 0.1 were selected. The low gamma value effectively generalized the model by spotting more significant patterns in the data, while the moderate C value avoided over fitting by maintaining tolerance for minor prediction errors. According to^25^, who emphasized the importance of gamma and C adjustments for the best model generalization in regression tasks, this approach adheres with SVR hyper-parameter tuning best practices.

Extreme Gradient Boosting (XGBoost): it a boosting-based ensemble machine learning algorithm widely used for classification, regression, and ranking tasks, and can be viewed as an advanced implementation of gradient boosting machines (GBMs)^27^. Similar to standard GBM, it builds an additive sequence of weak learners, where each subsequent learner is trained to correct the residual errors of the previous ensemble, implementing the boosting principle. A key advantage of XGBoost is its use of a second-order approximation of the objective function, which stabilizes and regularizes the loss, improves predictive accuracy, and accelerates convergence to an optimal solution^26^. Additional enhancements, including efficient parallelization, sparse-aware computation, and support for distributed training, make XGBoost substantially faster and more scalable than conventional GBM implementations, particularly on large, high-dimensional datasets. The hyperparameter configuration used have been detailed in Table 12.

#### Spatial K-fold cross-validation

Spatial k-fold cross-validation (5 folds) was implemented using KMeans-clustered spatial blocks on pixel coordinates to mitigate autocorrelation in the 1.5 million-sample GIS raster dataset, ensuring geographic separation between training (80%) and validation (20%) folds for unbiased R^2^, RMSE, and MAE estimation^47^. Random Forest, XGBoost, and SVR models were evaluated across spatially independent folds, with performance metrics averaged to validate predictive generalization for wind farm suitability mapping across North Shewa of Amhara region in Ethiopia.

#### Statistical evaluation metrics

Widely accepted statistical evaluation metrics were applied in this research to objectively compare the predictive performance of the selected machine learning models. Quantitative measures used to assess the performance, accuracy, and overall effectiveness of machine learning models are statistical evaluation metrics^37,38^. These metrics help in comparing various algorithms or model configurations and offer objective criteria for assessing how well a model generalizes to unknown data. Statistical evaluation metrics used in this research are:

Root Mean Squared Error (RMSE): The square root of mean squared error can be used to calculate the average magnitude of the error between the actual and predicted values, as Eq. (15)^37^ illustrates. A lower RMSE indicates a better predictive model.

15 $$RMSE = \sqrt {\frac{1}{{\mathrm{n}}}\mathop \sum \limits_{{{\mathrm{i}}=1}}^{{\mathrm{n}}} {{\left( {{{\mathrm{P}}_{\mathrm{i}}} - {{\mathrm{O}}_{\mathrm{i}}}} \right)}^2}}$$

Where ${{\mathrm{O}}_{\mathrm{i}}} -$Actual value (observed value) of the i-th observation and ${{\mathrm{P}}_{\mathrm{i}}} - {\mathrm{Predicted~value~}}\left( {{\mathrm{forecast~value}}} \right){\mathrm{for~the~}}{\mathbf{i}} - {\mathrm{th~observation~from~the~mode}}.$

Coefficient of Determination (R2): In a model with a range of 0 to 1, it is the percentage of the dependent variable’s variance that can be accounted for by the independent variable^37^. The model performs better when the indicated values are higher. The formula for R2 is Eq. (16)^36^.

16 $${\mathrm{R}}^{2} = 1 - \frac{{\mathop \sum \nolimits_{{{\mathrm{i}} = 1}}^{{\mathrm{n}}} \left( {{\mathrm{O}}_{{\mathrm{i}}} - {\mathrm{P}}_{{\mathrm{i}}} } \right)^{2} }}{{\mathop \sum \nolimits_{{{\mathrm{i}} = 1}}^{{\mathrm{n}}} \left( {{\mathrm{P}}_{{\mathrm{i}}} - \mathop {\mathrm{P}}\limits^{ \vee } } \right)^{2} }}$$

Where $\mathop {\mathrm{P}}\limits^{ \vee }$-Mean value of actual response

To guarantee reliability, the raw data was rigorously pre-processed before these metrics were applied.

Mean Absolute Error (MAE): MAE measures the average magnitude of errors in a set of predictions, without considering their direction. It represents the average absolute difference between the actual observed values and the predicted values from the model, and it quantifies prediction accuracy where lower values indicate better performance. The formula for MAE is given by (17):

17 $$MAE = \left( {\frac{1}{n}} \right) \times \sum \left| {{{\mathrm{O}}_{\mathrm{i}}} - {{\mathrm{P}}_{\mathrm{i}}}} \right|$$

## Result analysis

The results of integrated FAHP-DEA-ML framework applied to strategic wind farm site selection have been presented in this section. The sequential results are organized to reflect methodological workflow: FAHP results, DEA efficiency score, machine learning classification results and mapping and validation of the results.

### FAHP model result and comparison with AHP

**Fig. 6:**
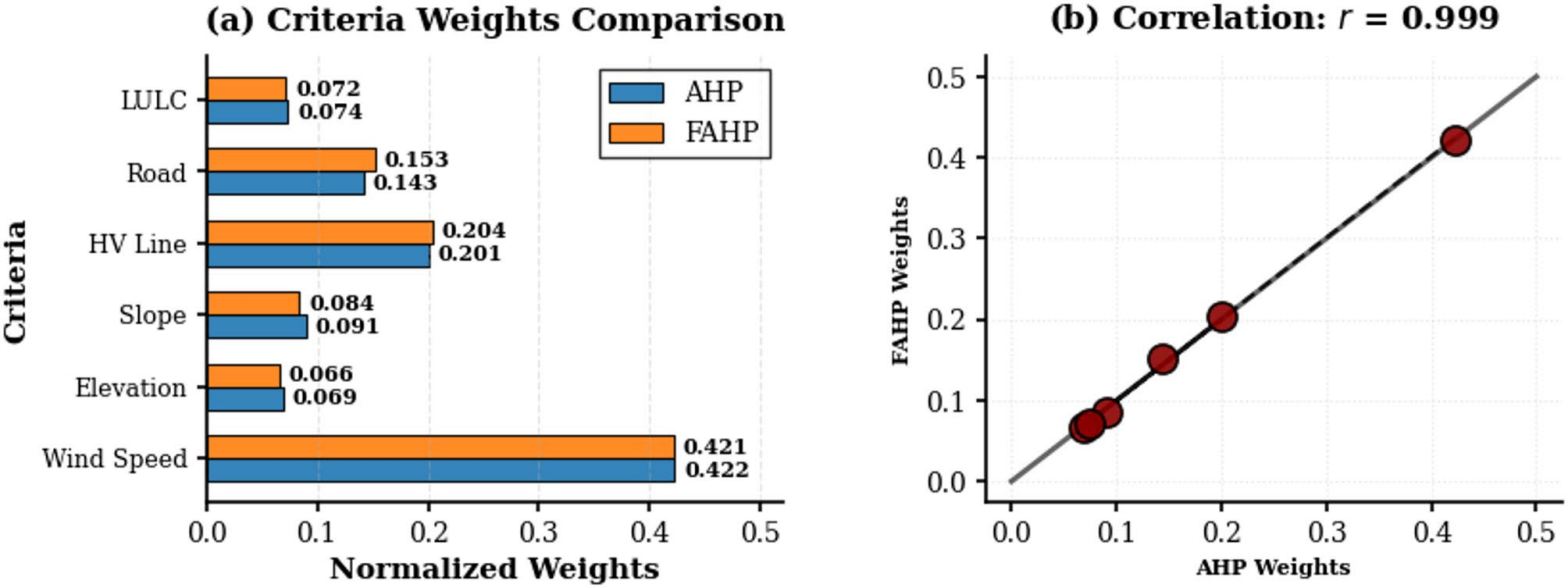
Fuzzy AHP-derived criterion weights for wind-farm site selection in North Shewa Zone.

The FAHP results for wind farm site selection are consistent and reliable, with a consistency ratio of 0.0175 with CR < 0.1 that indicates experts judgment are coherent. As it can be shown in Fig. 6 wind speed is the dominant factor with the weight of 0.4211, while proximity to high-voltage transmission lines at the weight of 0.2040. Substantial emphasis also given to main roads with 0.1528 weight factor. This shows that resource quality and grid costs jointly drive site suitability of wind farms. In contrast, elevation (0.0655), slope (0.0844), and LULC (0.0721) play secondary but non-negligible roles. It plays mainly constraining terrain feasibility and land-use compatibility rather than determining the primary ranking of wind farm site candidate locations.

The comparative analysis of AHP and FAHP for wind farm site selection criteria in Ethiopia’s Amhara region reveals excellent methodological consistency (CR = 0.0175 < 0.1, Pearson *r* = 0.989), with both approaches confirming wind speed (C1) as dominant at ~ 42% weight while FAHP appropriately elevates infrastructure proximity criteria (HV transmission C4 + 29%, roads C5 + 55%) to better capture expert uncertainty. This alignment validates the fuzzy pairwise judgments while highlighting FAHP’s advantage for handling linguistic vagueness inherent in regional expert elicitation, making it preferable for subsequent DEA efficiency screening and ML suitability mapping targeting North Shewa’s validated wind corridors.

**Fig. 7:**
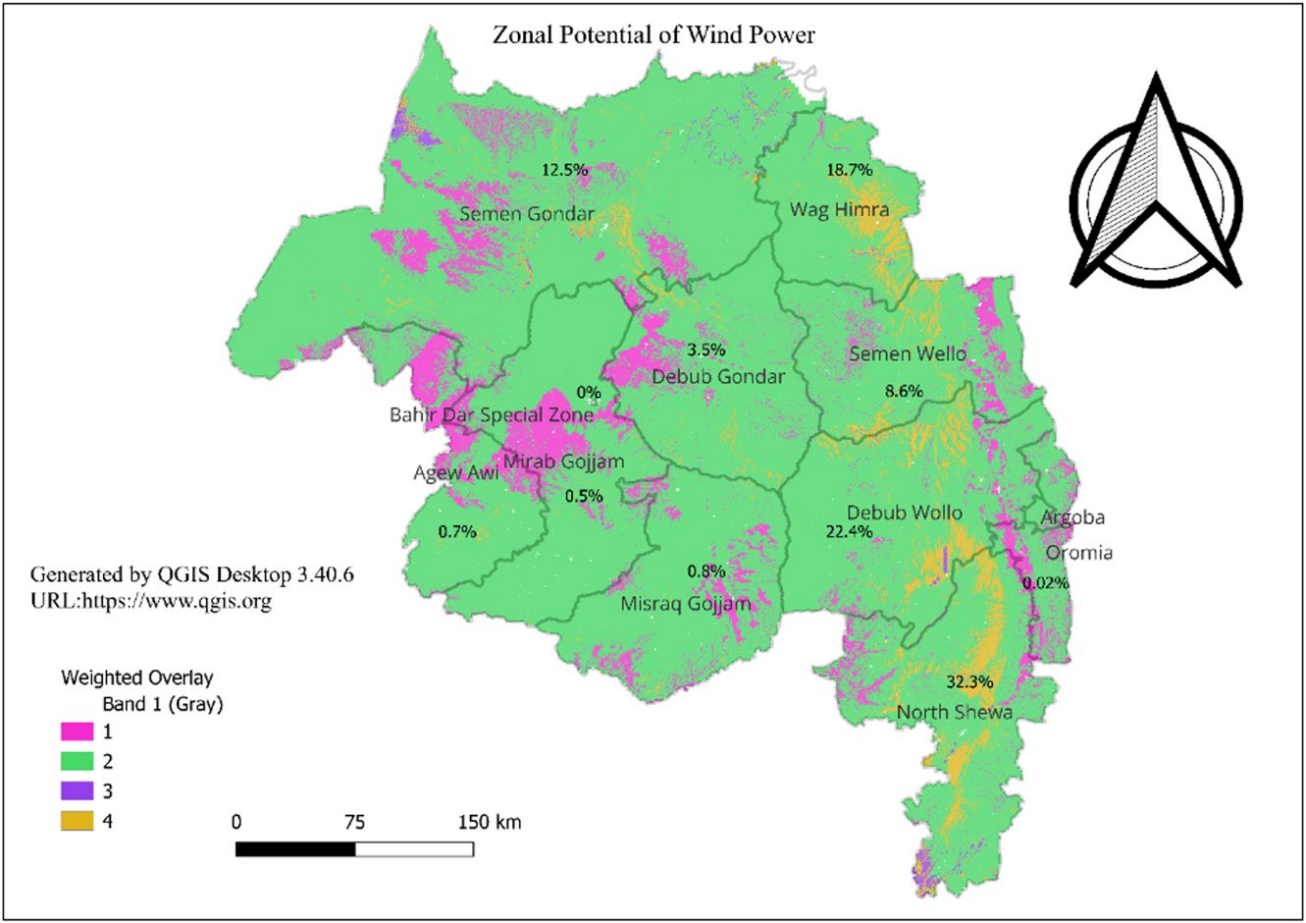
Wind power plant suitability map of Amhara region.

The map in Fig. 7 illustrated the spatial distribution of wind energy potential suitability across Ethiopia’s Amhara region. The zonal wind power potential map for Amhara region shows a highly uneven spatial distribution of suitable land among administrative zones, with North Shewa (32.3%), South Wollo (22.4%) and Wag Himra (18.7%) emerging as the dominant contributors to the regional resource together counted more than half of the identified high-potential land as most suitable. Intermediate zones, including North Gondar (12.5%), and North Wello (8.6%), provide substantial development opportunities and could support a second tier of wind projects that complement the primary investment focus on North Shewa, South Wollo and Wag Himra. The spatial clustering of high-potential pixels along the eastern escarpment and parts of the northern western highlands is consistent with known wind corridors in Ethiopia.

Figure 6 also demonstrated that 4.66% areas of the region (orange) is optimally suitable for wind energy development and represented as 4. This area has flat to moderately elevated terrains with high wind speed and accessible infrastructure. 0.65% of the region(blue) were potentially suitable with moderate development effort while most of the region coloured in green (84.35%) have noticeable constraints such as topographic and land use restrictions as well as infrastructure and wind speed limitations. The rest 10.33% of the region represented in 1(Pink) has environmental and physical constraints which are listed in the criteria of this study to make wind energy development infeasible.

### DEA model result

The DEA and AHP results in Table 13 show that only three zones such as North Shewa, North Wollo, and North Gondar were operated on the efficient frontier under both CCR and BCC models (scores = 1), with scale efficiency equal to 1 and SBM scores of 1 to confirm that their current input–output combinations are fully efficient. North Shewa and North Wollo coincide with the highest AHP-derived suitability shares. Rank change occurs between Wag Himera and North Gondor. This indicated that there is strong agreement between the DEA efficiency analysis and the multi-criteria resource suitability assessment. In contrast, zones such as Argoba and Oromia exhibit low CCR and scale efficiency scores of approximately 0.50 and 0.47, respectively and negligible SBM and AHP values. This implies that both have limited wind-suitable area and relatively inefficient use of their inputs and these DMUs would require substantial proportional reductions in inputs to approach the efficient frontier. Intermediate performers such as North Wello, East Gojjam, and West Gojjam have relatively high CCR scores (0.77–0.97) and small slack-based inefficiencies but lower AHP shares than the top three zones. It suggests that while they use their current resources reasonably efficiently, their contribution to regional wind potential is more modest, and they may be better suited for second-phase or medium-scale developments rather than immediate star projects.

**Table 13:** DEA–AHP efficiency scores and overall suitability ranking of administrative zones for wind farm development.

DMU	CCR	BCC	Scale_Eff	SBM	AHP	Overall ranking
Agew Awi	0.6557	0.9958	0.6584	0.0044	0.74	
Argoba	0.4987	0.9964	0.5005	0.0001	0.01	
Bahir Dar Special Zone	0.7458	1.0000	0.7458	0.0000	0.00	
South Gondar	0.7459	0.9960	0.7489	0.2055	3.55	
**South Wollo**	**1.0000**	**1.0000**	**1.0000**	**1.0000**	**22.35**	**2**
West Gojjam	0.8055	0.9966	0.8083	0.0396	0.55	
East Gojjam	0.9125	0.9981	0.9142	0.0654	0.78	
**North Shewa**	**1.0000**	**1.0000**	**1.0000**	**1.0000**	**32.25**	**1**
Oromia	0.4706	0.9973	0.4718	0.0014	0.02	
**North Gondar**	**1.0000**	**1.0000**	**1.0000**	**1.0000**	**12.46**	**3**
North Wello	0.7661	0.9958	0.7694	0.3077	8.61	
Wag Himra	0.9737	0.9978	0.9759	0.2633	18.70	

Significant values are in [bold].

**Fig. 8:**
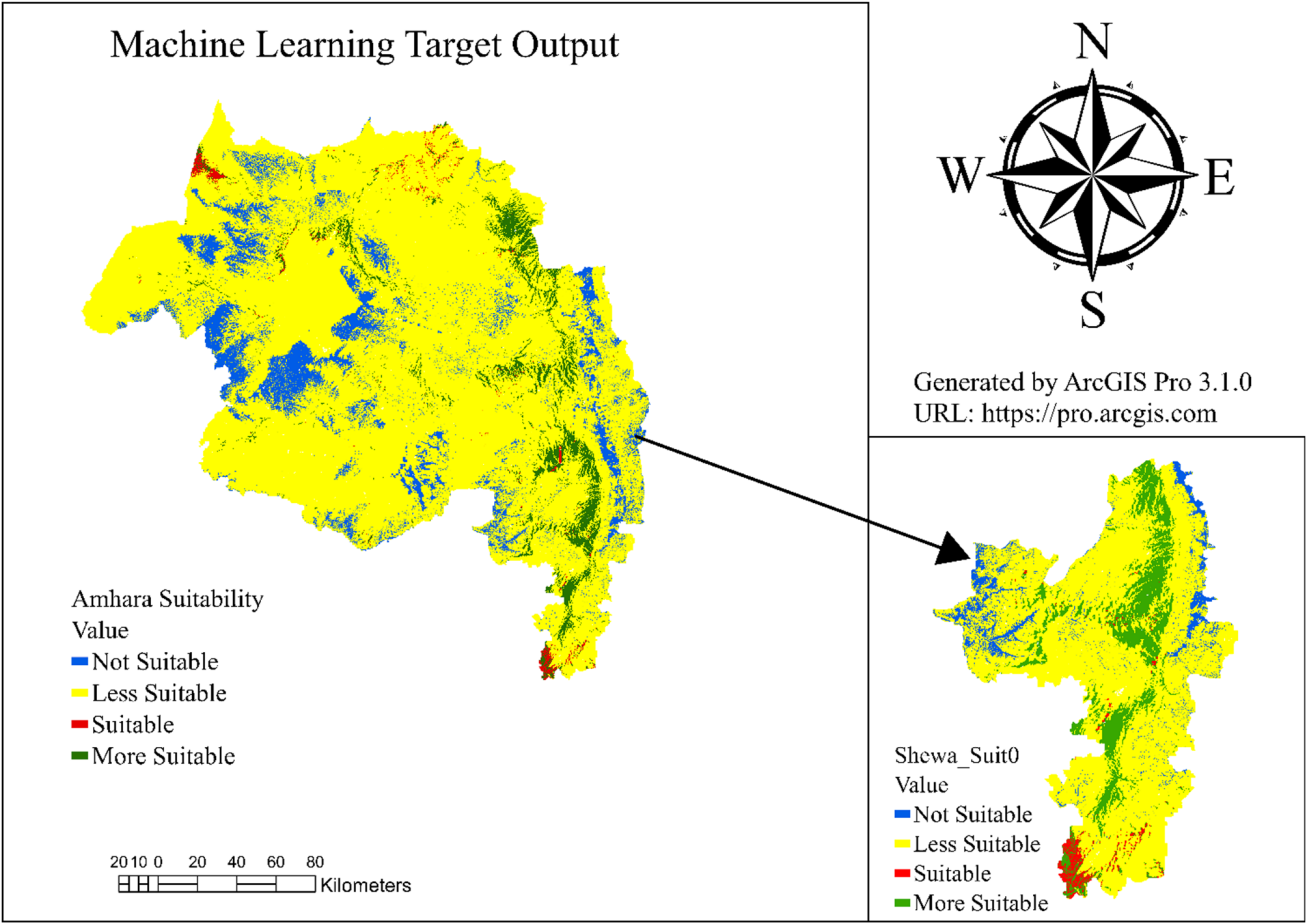
FAHP-DEA Suitability map of North Shewa.

Thus, the best performer zone of the region based on two consecutive results (FAHP and DEA) is North Shewa and the suitability map has been clipped out as depicted in Fig. 8.

**Fig. 9:**
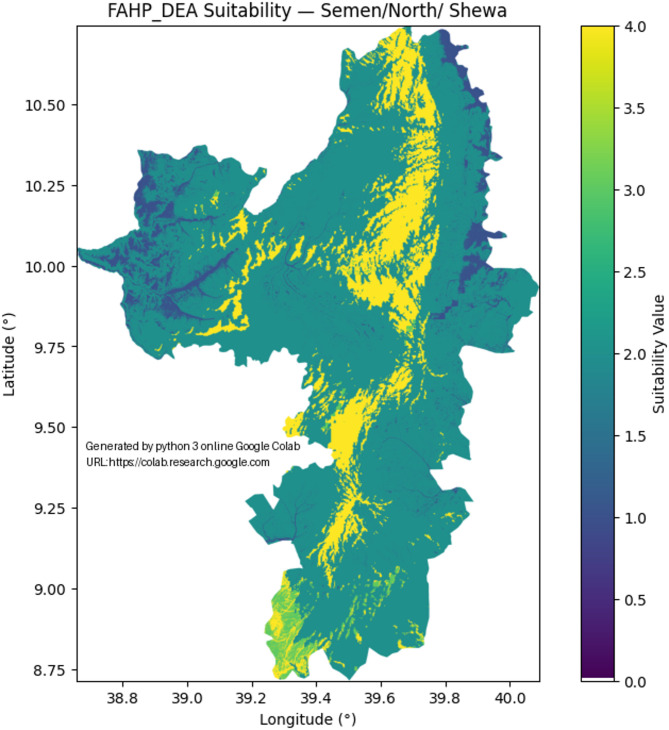
Machine-learning target suitability map for the Amhara Region and clipped suitability output for the best-performing North Shewa Zone derived from combined FAHP and DEA results.

The integrated FAHP–DEA framework pictured in Fig. 9 identifies North Shewa as a premier wind energy development corridor in Amhara Region that combined high technical suitability with strong economic relevance. FAHP results show that wind speed and grid/road proximity dominate the criteria hierarchy to ensure that areas selected as “suitable” simultaneously maximize energy yield and minimize infrastructure costs. DEA then confirms that only a subset of zones particularly North Shewa operate on the efficiency frontier and this indicates that the zone converts available land, wind resource, and accessibility into potential energy output more effectively than other zones in the region. The suitability map screened by FAHP_DEA in Fig. 9 has been processed for data missing and outliers using geopandas, a python library for geospatial application, before pipelined into ML.

### Validation of FAHP_DEA method

North Shewa, identified in the proposed FAHP–DEA framework as the top-priority zone (Rank 1, CCR = 1.0) with 32.25% of the regional suitable area, is strongly substantiated by five independent studies shown in Table 14 spanning national planning, academic research, and professional engineering assessments. The national wind master plan by Hydrochina Corporation locates three major wind farm sites—Debre Sina (50 MW, 9.0 m/s), Chacha (100 MW, 8.0 m/s), and Debre Birhan (100 MW, 8.0 m/s)—within this administrative zone of the region to provide 250 MW of installed capacity collectively^45^. Authors in^43^ (2023) conducted ground-based feasibility assessments at seven sites across North Shewa over 19 years (2000–2019) to confirm annual average wind speeds of 3.5 m/s at 10 m height, extrapolating to 8.5 m/s at 50 m and 12.9 m/s at 100 m hub height, with wind power densities reaching 945.2 W/m^2^ and energy densities of 11,417.1 kWh/m^2^/year at 100 m turbine height. Most significantly, SgurrEnergy Ltd. (2014) performed a bank-grade energy yield assessment for a proposed 100 MW Debre Berhan wind farm (67 × 1.5 MW turbines, 85 m hub height) located approximately 35 km southwest of Debre Berhan town in North Shewa. Based on 18 months of meteorological measurements (October 2012–May 2014) at the Sembo 1202 mast with a coordinate of 9.416°N, 39.341°E. At this site a long-term mean wind speed has been predict to 6.68 m/s at 85 m hub height and energy yield of 245.2 GWh/annum at capacity factor of 27.9%^44^. Additionally, in^46^ (2020) analysed five years (2014–2018) of daily wind speed data measured at 2.5 m height in Debre Berhan to report monthly mean wind speeds ranging from 3.95 m/s in August to 5.59 m/s in February at 50 m height, with monthly mean wind power densities of 39.33–111.60 W/m^2^^46^. Extrapolating this to standard height would confirm the site’s suitability for wind turbine installations in the areas. This framework’s prioritization of North Shewa is also corroborated by Javid sharifi et al., whose 2025 academic research measured 50 m wind speeds yielding 200 MW capacity at Sela Dengay within the zone^48^. This empirical validation through ground-truth anemometry complements our pixel-level RF predictions (R²=0.8153, 5-fold cross-validation) and DEA efficiency frontier analysis, confirming North Shewa as Amhara’s premier wind development corridor across independent methodologies—fuzzy MCDM, nonparametric efficiency and ensemble ML.

**Table 14:** Multi-source validation of FAHP-DEA framework zone rankings against independent national and engineering studies.

Source	Type	Key finding	North Shewa validation
Ayalke & Şişman (2022)^21^	Academic Research	Eastern and Western part of the region is suitable for wind farm	Published in Peer-Reviewed Journal
Hydrochina Master Plan for Ethiopia^44^	National Planning	3 sites located in proposed zone: 250 MW capacity Project sites: Debre Sina, Chacha, Debre Birhan	National Report and Published in Peer-Reviewed Journal
Feleke et al. 2023 - and published Peer-Reviewed Journal^42^	Academic Research	12.9 m/s @ 100 m (extrapolated), 945 W/m^2^	Comprehensive long-term validation in North Shewa
SgurrEnergy 2014 - Bank- grade feasibility study, 100 MW project^43^	Engineering Assessment	100 MW wind farm feasibility, 6.68 m/s at 85 m, 245 GWh/year, 27.9% capacity factor	Bank-grade feasibility study, 100 MW project for locations in the zone
Woldegiyorgis et al. (2020)^45^	Academic Research	5.59 m/s @ 50 m (Feb peak), 111.6 W/m^2^	Published validation in Applied J. Env. Eng. Sci.

**Fig. 10:**
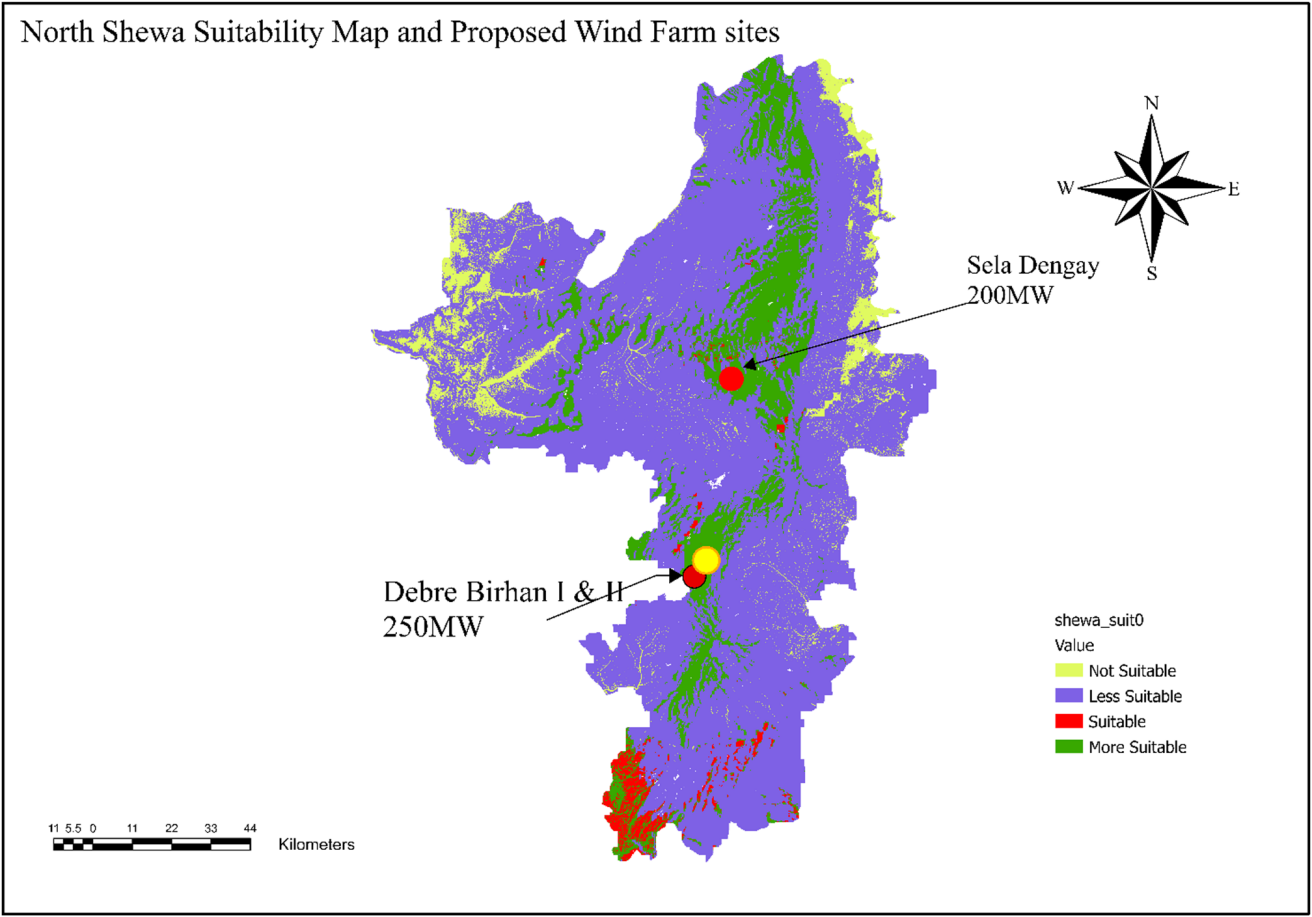
Proposed Wind Farm sites in North Shewa and Suitability corridor.

Figure 10 demonstrates comprehensive alignment with Ethiopia’s Ministry of Finance(MOF) and Ethiopian Electric Power(EEP) Public-Private Partnership(PPP) pipeline, where the model’s high-suitability corridors (RF = 0.8153) precisely capture the Debre Berhan Wind Power Projects—one of five wind PPP initiatives approved since 2020 alongside Aysha II and Diday of Somali region, Adigala of Afar region, and Dire Dawa—confirming spatial concordance with national 10-year commercialization strategy. North Shewa’s DEA-frontier status encompasses MoF’s Debre Berhan allocation within the framework’s 1,698 km^2^ priority delineation, validating predictive accuracy across both state-owned EEP planning and federal PPP frameworks for Ethiopia’s wind expansion targets.

Furthermore, the selection of criteria in the FAHP–DEA–ML framework is validated by the established criteria set used in the Amhara Region GIS–BWM study^21^. The dominance of wind speed in the weighting structure of this study confirms consistency with previously validated site selection models applied in the same geographic context. The spatial distribution of highly suitable areas identified in this study corresponds to the eastern zones of the Amhara region reported in the GIS–BWM analysis. This spatial similarity supports the external validity of the proposed FAHP–DEA–ML framework.

These seven sources—encompassing the proposed FAHP-DEA framework, the national master plan (3 sites, 250 MW), academic measurements including 19-year dataset, ministry of finance PPP pipelines and professional engineering validation of bank-grade feasibility study—collectively confirm that the zone prioritized by the FAHP–DEA model as Rank 1 coincides with the empirically most wind-rich and commercially viable development corridor in the Amhara Region and it also confirm the methodological robustness.

### Machine learning results

The machine learning 5-fold stratified cross-validation performance evaluation in Table 15 indicates that Random Forest achieved optimal classification and regression performance among the three tested models for selecting optimal placement of wind farm in North Shewa zone partitioning the 31-million-pixel dataset into five mutually exclusive folds while preserving class distribution ratios. Random Forest (RF), XGBoost, and SVM models underwent comprehensive evaluation that results mean F1-scores of 0.9207 (RF), 0.9277 (XGBoost), and 0.8861 (SVM), alongside accuracies of 0.9237 (RF), 0.9297 (XGBoost), and 0.8945 (SVM). XGBoost demonstrated superior classification performance, while RF excelled in regression metrics (RMSE = 0.2944, MAE = 0.2520, R²=0.8153) that confirms both ensembles’ suitability for operational wind farm suitability prediction within the integrated FAHP-DEA-ML framework. While XGBoost shows marginal classification gains (0.7%), Random Forest delivers superior regression performance and remains excellent in classification. This dual superiority makes RF the optimal choice for comprehensive wind farm suitability modelling. XGBoost performed slightly lower performance with RMSE 0f 0.3234, MAE of 0.1487 and R2 of 0.7831 but still maintained reasonably strong predictive power. This also suggested that it still remains a viable alternative. Support Vector Machine showed the least performance (RMSE = 0.3599, MAE = 0.1610, R2 = 0.7360 F-score = 0.8861, Accuracy = 0.8945) and it tends to generalize less effectively than ensemble methods.

**Table 15:** Performance of candidate machine-learning models K-Fold Cross-Validation for wind-farm suitability prediction.

Fold	RF			XGBoost			SVR		
	RMSE	MAE	*R*²	RMSE	MAE	*R*²	RMSE	MAE	*R*²
1	0.2981	0.2542	0.8143	0.3234	0.1498	0.7831	0.3687	0.1621	0.7360
2	0.2918	0.2501	0.8172	0.3169	0.1474	0.7862	0.3650	0.1597	0.7391
3	0.2943	0.2513	0.8155	0.3190	0.1483	0.7845	0.3668	0.1607	0.7373
4	0.2933	0.2517	0.8158	0.3184	0.1485	0.7849	0.3660	0.1609	0.7377
5	0.2946	0.2526	0.8139	0.3197	0.1493	0.7818	0.3676	0.1617	0.7346
Mean	0.2944	0.2520	0.8153	0.3195	0.1487	0.7841	0.3668	0.1610	0.7369
F1-score	0.9277			0.9207			0.8861		
Accuracy	0.9297			0.9237			0.8945		

**Fig. 11:**
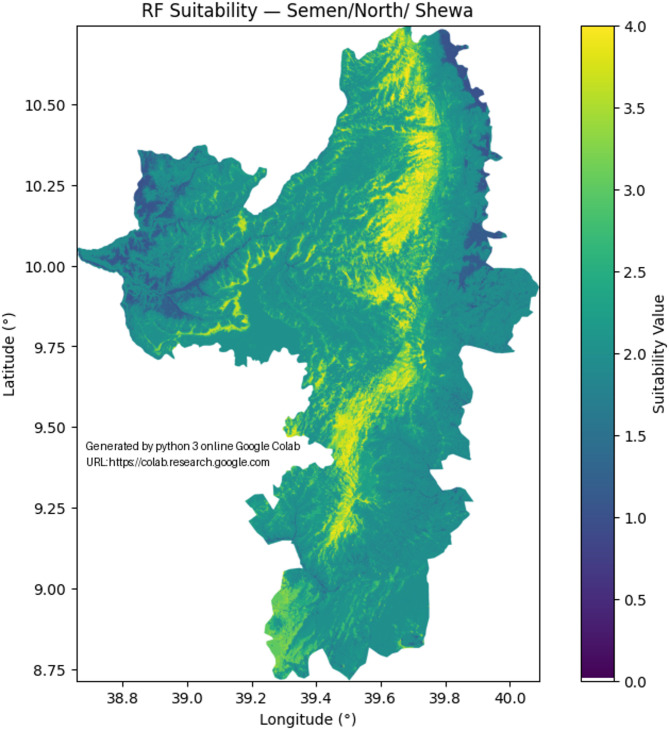
Random-forest–derived wind-farm suitability map for the best-performing North Shewa Zone.

Training the Random Forest model on 1.5 million pixels sampled from the 31-million-cell suitability surface successfully reproduced the FAHP–DEA zones pattern in order to tackle computation burden while adding finer local detail. The RF-based map for North Shewa in Fig. 9 shows a continuous central corridor of high suitability values that closely follows the main ridge and escarpment that matches the high-suitability belt identified by the original FAHP–DEA weighted overlay in Fig. 8. At the same time, the ML output reveals additional micro-scale variation within this corridor and along valley edges to reflect how the RF model learns non-linear interactions among wind speed, terrain, accessibility, and land use similar to other ML siting studies. Overall, the strong spatial agreement between both maps such as Figs. 9 and 11 indicates that FAHP–DEA provides a robust, interpretable baseline for multi-criteria prioritization, while the Random Forest generalization enables high-resolution suitability mapping across the full 31-million-cell domain without re-running the full MCDM pipeline.

### Area of potential zones

**Fig. 12:**
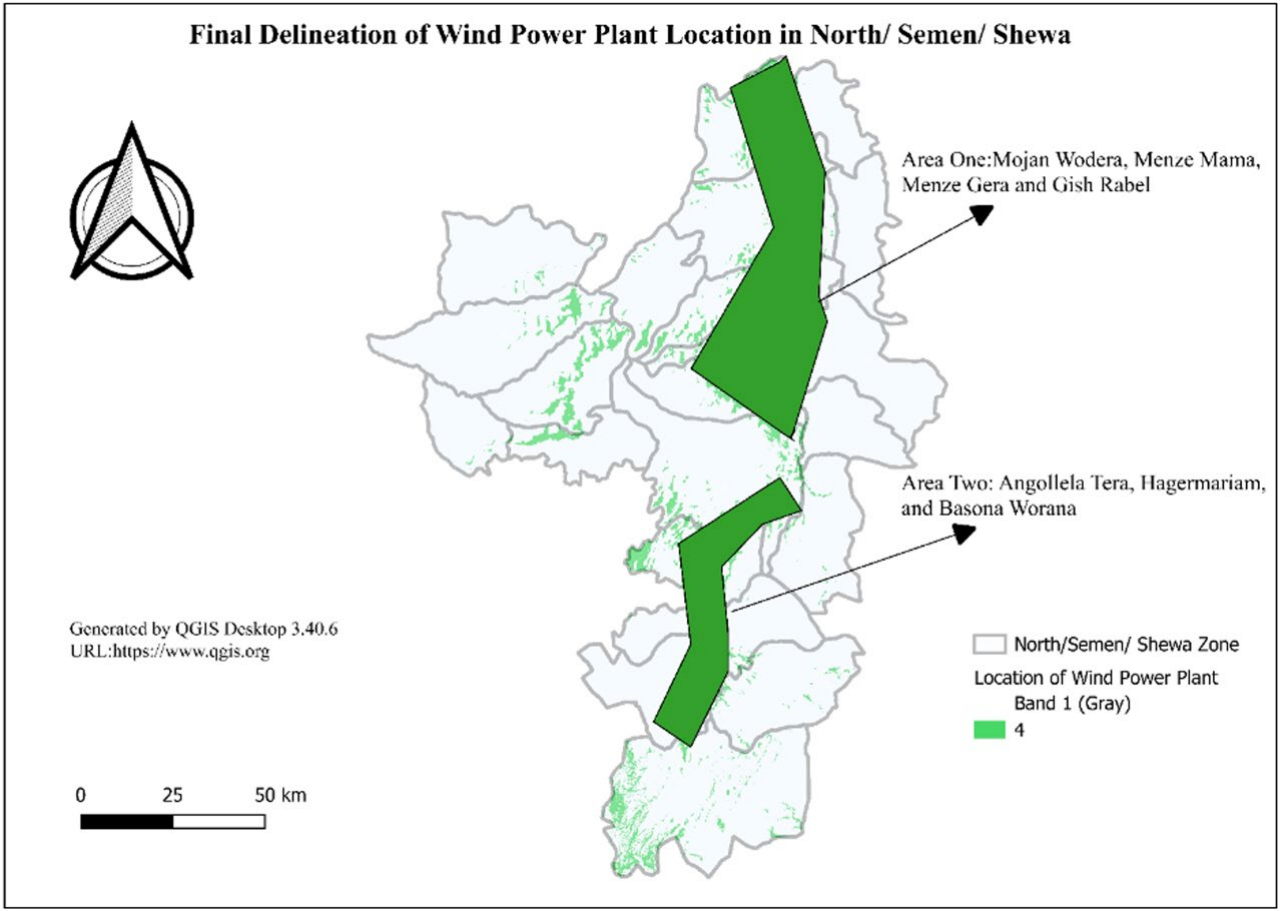
Final delineation of candidate wind power plant locations within the best-performing districts of North Shewa Zone.

the final delineation map for North Shewa described in Fig. 12 identifies two continuous, corridor-like polygons as the most suitable locations for large-scale wind power plant development that synthesizes the outputs from the FAHP–DEA and RF suitability analyses into concrete planning zones. Area One that extends across Mojan Wedera, Menze Mama, Menze Gera and Gish Rabel has followed the main high-suitability ridge in the northern part of the zone, where strong wind resource, favourable topography, and good access to existing infrastructure coincide with earlier zonal and pixel-level suitability patterns. Area Two that spans Angolela Tera, Hagermariam and Basona Worena district in the southern corridor which represents a second strategic cluster where suitability remains high and relatively contiguous for staged expansion or multiple wind farm projects rather than isolated and scattered turbines. Together, these delineated areas translate abstract suitability scores into spatially coherent development blocks that can directly support detailed layout design, environmental impact assessment, and grid integration studies for priority wind investments in North Shewa.

The district reclassification demonstrated in Table 16 shows that all selected districts in North Shewa contain substantial tracts of land in the “More suitable” and “relatively suitable” classes. In Area one (northern blocks) together contribute 1,217,303 suitable pixels that corresponds to about 1,095.6 km² of highly suitable land. In Area two (southern blocks) add another 669,537 suitable pixels that estimated to roughly 602.6 km² to North Shewa as another development corridor. The total of about 1,698 km^2^ of suitable land in North Shewa from both corridors provides a strong quantitative justification for prioritizing this zone in regional or national wind planning.

**Table 16:** Suitability classification of grid cells for wind-farm development in districts of North Shewa Zone and corresponding suitable-area estimates.

Districts in North Shewa	Not sutable	Less suitable	Suitable	More suitable	Percentage (More Suitable)
Angolelana Tera	5189	579,494	8570	323,510	13.85%
Basona Worena	20,519	1,272,763	10,775	207,683	22.84%
Hagere Mariam	19,814	601,363	650	138,344	11.49%
Suitable cells count				669,537	35.48%
Southern Block -Area 2 (km^2^)				602.5833	
Gishe Rabel	6810	470,269	238	284,923	11.52%
Menz Gera Midir	4244	946,201	341	289,421	18.74%
Menz Mama Midir	2033	327,629	4671	390,354	10.95%
Mojan Wedera	10,287	432,004	7273	252,605	10.61%
Suitable cells count				1,217,303	64.52%
Northern Block-Area 1 (km^2^)				1095.5727	
Total area (km^2^)				1698.156	

Southern Block Suitability: The Southern Block encompasses three districts with a total suitable area of 602.58 km² (669,537 suitable cells) to represent 35.48% of the zone’s total suitable area. Basona Worena demonstrates the highest suitability percentage at 22.84%, followed by Angolelana Tera at 13.85%. Hagere Mariam exhibits the lowest suitability at 11.49% which indicates more restrictive site conditions. Basona Worena emerges as the primary candidate for wind farm development within this block to offer both the highest suitability percentage and substantial contiguous suitable areas.

Northern Block Suitability: The Northern Block comprises four districts with a total suitable area of 1,095.57 km² (1,217,303 suitable cells) which represents 64.52% of the suitable land within North Shewa Zone. This indicates that, although only roughly one-third of the zone is appropriate for wind-farm development, nearly two-thirds of that limited suitable land is concentrated in the Northern Block to underscore its strategic importance. Within this block, Menz Gera Midir contributes the largest share of suitable area (18.74%), followed by Gishe Rabel (11.52%), Menz Mama Midir (10.95%), and Mojan Wedera (10.61%) to form a compact cluster of comparatively favourable locations. The dominance of the Northern Block within the constrained 32.3% suitable fraction suggests that this area combines relatively strong wind resources, favourable terrain, and fewer exclusion constraints under the Fuzzy AHP evaluation to make it the priority focus for wind-farm planning at the zonal scale.

## Discussion

This section provides discussion of integrated FAHP-DEA-ML framework to examine the synergistic combination of methods in wind farm site selection. The discussion is organized into three themes: Integration of FAHP and DEA, machine learning performance and spatial generalization and policy and practical implementation.

### Integration of FAHP and DEA

The proposed three-stage framework successfully addresses critical gaps in conventional wind farm site selection methodologies by combining subjective expert knowledge, objective efficiency analysis, and data-driven predictive modelling. While previous studies have employed FAHP for uncertainty handling^10–17^ DEA for efficiency screening^20^, and otherwise recent works have explored ML-GIS integration^26–29^, no prior research has unified all three components to simultaneously manage vagueness through fuzzy logic, reduce subjectivity via non-parametric efficiency analysis, and enable spatial generalization through RF. The FAHP stage effectively captured expert uncertainty through triangular fuzzy numbers to get a consistent pairwise comparison matrix with CR of 0.0175 that prioritizes wind speed at the weight of 0.4211 and grid proximity weight of 0.2003 while appropriately constraining terrain factors. This weighting structure aligns with established wind energy planning principles that emphasize resource quality and economic accessibility over secondary topographic considerations^21^. However, the static nature of FAHP-derived suitability maps limits their ability to discriminate among candidate zones with similar weighted scores.

The DEA component addresses the limitations encountered by FAHP by treating each administrative zone as a DMU and computing relative technical efficiency under multiple input-output scenarios. The agreement between DEA efficiency rankings of North Shewa achieving frontier efficiency with CCR = BCC = SBM = 1.0 and FAHP suitability shares of 32.25% for North Shewa demonstrates that the most wind-rich zone and also utilizes their infrastructure, terrain, and demand conditions most efficiently. This dual validation strengthens confidence in the final prioritization and reduces the risk of selecting sites that appear suitable on paper but prove inefficient in practice. FAHP-DEA ranking divergence for North Gondar and Wag Himra stems from DEA’s objective penalization of low population density and infrastructure remoteness for Wag Himra, while FAHP favours their wind resource and terrain. This highlights the framework’s ability to balance technical potential with practical efficiency.

### Machine learning performance and spatial generalization

The ML stage employs 5-fold cross-validation to extend zone-level DEA ranking to pixel-level suitability prediction across the 31-million-pixel Amhara study domain. Random Forest demonstrates optimal generalization mean RMSE = 0.2944, 0.2520, R^2^ = 0.8153, F1 = 0.9277 and accuracy = 0.9297), outperforming XGBoost and SVR by effectively capturing non-linear spatial interactions among FAHP-weighted criteria without overfitting. This cross-validated performance aligns with recent wind siting literature where ensemble methods excel over gradient boosting and kernel alternatives^24,29^ and parallels Chaibi et al.‘s XGBoost supremacy (R^2^ = 0.9982) for fuzzy-MCDM validation^46^. The RF model’s RMSE of 0.2981 corresponds to approximately 10% relative error on the normalized 1–4 suitability scale, which is acceptable given the inherent uncertainty in expert-weighted MCDM frameworks and the spatial heterogeneity of predictors.

Critically, the RF-derived suitability map for North Shewa reproduces the main FAHP-DEA high-suitability ridge while revealing finer-scale variation along valley edges and escarpment zones. This demonstrates that the ML model successfully learned the underlying spatial function embedded in the FAHP-DEA training labels and generalized it to unsampled locations and thereby enabling continuous suitability surfaces rather than discrete class boundaries. This also indicates that Random Forest model captured the complex, non-linear relationships between input variables (wind speed, slope, elevation, distance to grid, roads, and land use) and the composite FAHP-DEA suitability scores.

The model extrapolated learned patterns across the entire 31-million-cell feature space by training on 1.5 million sampled pixels and produce smooth gradients that reflect subtle spatial variations in site quality. This approach overcomes the limitation of traditional MCDM methods that resulted location-specific rankings without predictive capability and enables dynamic suitability mapping that can adapt to updated data.

The ML component also benefits from transfer learning principles: once trained on a well-characterized region, the model can be fine-tuned for adjacent areas with minimal additional training data. This transferability is valuable for data-scarce regions where FAHP expert panels or DEA input-output datasets may not be applicable.

The stratified sampling strategy of 1.5 million pixels from 31 million to be represented by 5% coverage achieved adequate representation of the full suitability distribution while maintaining computational feasibility. Stratification ensured proportional sampling across all suitability classes (unsuitable, suitable, most suitable) and spatial zones to prevent bias toward dominant high-density clusters.

### Policy and practical implications

The identification of 1,698 km^2^ of high-priority wind corridors in North Shewa provides practical guidance for Ethiopian energy planners and private developers. The two delineated areas (northern cluster spanning Mojan Wedera, Menze Mama, Menze Gera, and Gish Rabel; southern cluster covering Angolela Tera, Hagermariam, and Basona Worena) offer contiguous development blocks that support economies of scale in transmission infrastructure, construction logistics, and operational maintenance. Prioritizing these zones for detailed feasibility studies, environmental impact assessments, and grid integration plans would accelerate Ethiopian Amhara regional progress toward its renewable energy targets at less capital risk.

The framework’s ability to discriminate among candidate zones using objective efficiency metrics rather than relying exclusively on subjective weights enhances methodological transparency and analytical defensibility. Donor agencies, multilateral development banks, and private equity investors require such quantitative and replicable methodologies to justify renewable energy investments. The proposed FAHP-DEA-ML approach meets this requirement by producing independently verifiable suitability maps, quantitative efficiency scores, and validation statistics that that enable systematic updates. The delineation of two contiguous corridors (Area 1 = 1,095.6 km^2^, Area 2 = 602.6 km^2^) provides actionable planning zones that can directly inform environmental impact assessments, grid integration studies, and turbine layout optimization.

### Limitations

The Study acknowledged the following limitations:

- The FAHP weights are derived from a panel of five experts drawn from Ethiopian institutions, lacking global representation with potential regional biases. The criteria also limited to only six which exclude many important constraints. Sensitivity analysis for FAHP should also be limitation to show the robustness.
- The DEA models assume variable returns to scale but do not explicitly account for temporal dynamics such as seasonal wind variability.
- The ML training labels are derived from FAHP-DEA scores; the model inherits systematic biases present in the MCDM framework. The framework focuses on utility-scale onshore wind development and does not address offshore wind potential.

## Conclusion and recommendations

The integrated FAHP–DEA–ML framework successfully identified and prioritized wind farm sites in the Amhara region by coupling expert-based fuzzy multi-criteria mapping with objective efficiency analysis and data-driven prediction. FAHP-generated suitability maps revealed strong spatial clustering of high-potential areas along the northwest and eastern cliff, while DEA confirmed that operates on the efficient frontier under CCR, BCC, and SBM models that aligned well with high FAHP suitability share in the region. This concordance between FAHP and DEA indicates that the selected DEA-efficient zones not only exploit favourable wind and terrain conditions but also deliver higher socio-economic benefits per unit of technical and infrastructure input. The machine learning stage demonstrated that models such as Random Forest, SVM, and XGBoost can learn the patterns encoded in FAHP–DEA scores and extend suitability assessment to unsampled locations of region-wide planning tool and thereby transforming a static evaluation into a predictive. The proposed approach reduces judgment bias, improves robustness under noisy or incomplete data, and offers a reproducible workflow that can be adapted to other regions. Overall, the results indicate that the FAHP–DEA–ML framework provides a practically implementable and theoretically sound basis for strategic siting of wind farm development.

​Future research should extend the current framework by incorporating spatiotemporal dynamics and deep learning architectures to jointly model seasonal wind variability, long-term climate trends, and evolving demand patterns at finer spatial and temporal resolutions. Expanded panel of expert including international wind energy consultants, financial analysts, and environmental impact specialists would strengthen the representativeness and reduce regional bias. ​On the machine learning side, exploring other machine learning techniques such as federated learning and transfer learning could allow models trained in data-rich contexts to be adapted to data-scarce regions. Coupling the FAHP–DEA–ML suitability outputs with electrification planning and power system expansion models such as OnSSET would also enable joint planning of generation, transmission and thereby moving from site-level ranking to system-level investment roadmaps aligned with SDG7 and national electrification targets.

## Data Availability

The datasets used and/or analyzed during the current study are available from the corresponding author on reasonable request.
